# Understanding In Vivo Fate of Nucleic Acid and Gene Medicines for the Rational Design of Drugs

**DOI:** 10.3390/pharmaceutics13020159

**Published:** 2021-01-26

**Authors:** Shintaro Fumoto, Tsuyoshi Yamamoto, Kazuya Okami, Yuina Maemura, Chisato Terada, Asako Yamayoshi, Koyo Nishida

**Affiliations:** Graduate School of Biomedical Sciences, Nagasaki University, Nagasaki 852-8501, Japan; tsuyoshi.yamamoto@nagasaki-u.ac.jp (T.Y.); bb55619005@ms.nagasaki-u.ac.jp (K.O.); bb30116036@ms.nagasaki-u.ac.jp (Y.M.); bb55620011@ms.nagasaki-u.ac.jp (C.T.); asakoy@nagasaki-u.ac.jp (A.Y.); koyo-n@nagasaki-u.ac.jp (K.N.)

**Keywords:** liposome, lipid nanoparticle, plasmid DNA, oligonucleotide, transfection, mechanism

## Abstract

Nucleic acid and genetic medicines are increasingly being developed, owing to their potential to treat a variety of intractable diseases. A comprehensive understanding of the in vivo fate of these agents is vital for the rational design, discovery, and fast and straightforward development of the drugs. In case of intravascular administration of nucleic acids and genetic medicines, interaction with blood components, especially plasma proteins, is unavoidable. However, on the flip side, such interaction can be utilized wisely to manipulate the pharmacokinetics of the agents. In other words, plasma protein binding can help in suppressing the elimination of nucleic acids from the blood stream and deliver naked oligonucleotides and gene carriers into target cells. To control the distribution of these agents in the body, the ligand conjugation method is widely applied. It is also important to understand intracellular localization. In this context, endocytosis pathway, endosomal escape, and nuclear transport should be considered and discussed. Encapsulated nucleic acids and genes must be dissociated from the carriers to exert their activity. In this review, we summarize the in vivo fate of nucleic acid and gene medicines and provide guidelines for the rational design of drugs.

## 1. Introduction

The sequencing of human genome has enabled the revelation of the causes of disease at the genetic level [1]. Technologies such as antibodies and molecular targeting are also being developed [2,3,4]. Antibodies are effective when the target molecules circulate in the blood as a humoral factor or are accessible as cell surface antigens. However, the development of antibodies targeting intracellular molecules is relatively difficult. In such cases, nucleic acid therapy and gene therapy are promising options. Some chemically modified oligonucleotides with specific configurations, such as antisense and siRNA drugs, have managed to significantly overcome the biological barriers without any formulation agents, and have achieved remarkable success [5]. Oher nucleic acids such as decoy oligonucleotides [6], microRNA (miRNA) [7], aptamers [8] and circular RNA [9,10] are also available to target specific molecule(s). Gene therapy is also a promising and growing field of research because it allows not only the expression of proteins but also their inhibition [11,12]. mRNA delivery is one method for transient gene expression, and DNA delivery is desirable for long-term efficacy. However, to obtain gene expression from DNA, it is necessary to deliver DNA into the nucleus of the target cell, which is a difficult hurdle. The use of viral vectors allows for efficient gene delivery owing to the inherent ability of the viruses; however, there are concerns about their safety [13,14]. Development of non-viral gene delivery is preferable due to the advantage of industrial production. This review focuses on the in vivo fate of non-viral vectors, especially lipid-based ones, with the goal of providing the information necessary for the development of efficient and safe nucleic acid therapy and gene delivery strategies/systems. In addition, the necessary evaluation methods are discussed.

## 2. History of Non-Viral Nucleic Acid and Gene Delivery

Non-viral transfection of exogenous DNA has been studied for half a century [15]. Since the development of the method to prepare liposomes (thin lipid film hydration) by Bangham and colleagues [16,17,18], encapsulation of DNA in liposomes has been achieved [19,20]. Felgner et al. improved in vitro transfection efficiency using cationic liposomes containing synthetic cationic lipid *N*-[1-(2,3-dioleyloxy)propyl]-*N*,*N*,*N*-trimethylammonium chloride (DOTMA) [21] to form complexes with DNA (lipoplexes). It was demonstrated that a combination of DOTMA with helper lipid cholesterol was capable of transfection in vivo [22]. Cullis and colleagues have developed lipid nanoparticles (LNP) based on ethanol injection to encapsulate nucleic acids antisense and DNA [23]. Thereafter, ionizable lipids with rationally designed pKa values have contributed to improved delivery efficiency [24,25].

Naked plasmid DNA is an attractive in vivo transfection vector in animals. Wolff et al. found that direct injection of naked DNA and RNA into muscle produced efficient gene expression in vivo [26]. Subsequently, hydrodynamics-based in vivo transfection, which is a rapid large-volume injection in the vasculature, has been developed [27,28]. Hydrodynamics-based in vivo transfection is an efficient method for delivering foreign genes to the liver. However, the possibility for its clinical application is limited owing to the requirement of a large volume. Physical stimuli, such as electroporation [29,30,31,32] and sonoporation [33,34], can improve the transfection efficiency of naked plasmid DNA. Additionally, pressure-mediated transfection of plasmid DNA has been reported [35,36,37]. However, these methods require specific instruments and/or procedures (operations) for physical stimuli. More recently, Huang et al. developed an efficient nonhydrodynamic in vivo transfection method using lipid calcium phosphate nanoparticles (LCP) [38], but the preparation procedure of LCP (core production and subsequent lipid film hydration) is complicated when compared with ethanol injection for LNP.

Cationic polymers, such as polyethyleneimine (PEI) [39,40], dendrimers [41], polymeric micelles [42], and polyion complexes [43] are also promising. However, the concerns of accumulative toxicity and antigenicity must be resolved prior to clinical applications. In comparison, lipid-based carriers may be preferable in terms of accumulative toxicity and antigenicity. Recently, siRNA Patisiran-containing LNP (ONPATTRO^®^) and siRNA Givosiran-containing LNP (GIVLAARI^®^) have been clinically approved [44,45,46]. In January 2020, the outbreak of COVID-19 occurred [47], and the worldwide pandemic is currently continuing. Surprisingly, in the same year, the use of two mRNA-containing LNP-based vaccines against SARS-CoV-2 started in the USA based on the Emergency Use Authorizations from the Food and Drug Administration (FDA) [48,49]; thus, LNPs enable very rapid development of mRNA vaccines. Lipid-based carriers are an attractive and versatile platform to deliver not only siRNA, but also mRNA and plasmid DNA. Many clinical trials are ongoing using not only LNPs, but also other vectors such as PEI, as well as naked mRNA, plasmid DNA, and chemically modified antisense oligonucleotides (ASOs) [5,12,44,45,50,51,52].

Exosomes, a kind of extracellular vesicles, are known to contain nucleic acids such as mRNA and miRNA; thus, they are thought to be attractive delivery vehicles [53,54,55,56]. Recently, Yamayoshi et al. have designed a unique system, ExomiR-Tracker, for delivery of anti-miRNA oligonucleotide to target cells using anti-exosome antibodies [57].

**Figure 1:**
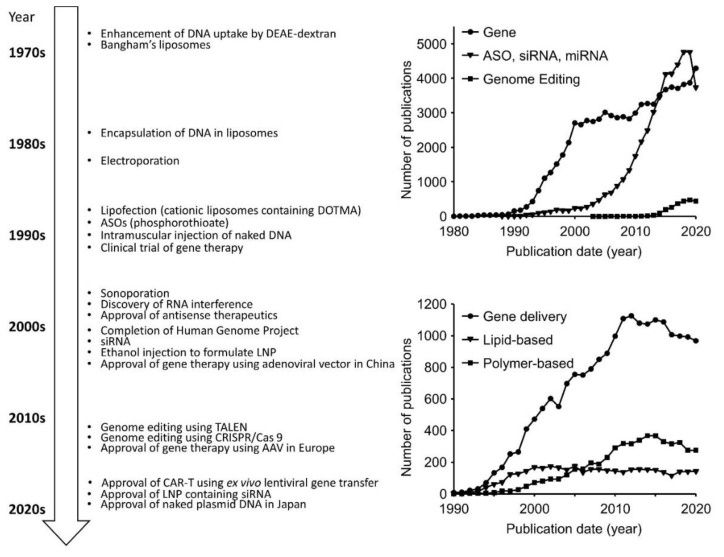
History of nucleic acid and gene medicines. The number of publications were searched via PubMed on 18 January 2021. Search queries: for “Gene”, (“Gene therapy” or “gene delivery”); for “ASO, siRNA, miRNA”, ((“siRNA” or “miRNA” or “microRNA” or “antisense oligonucleotides”) and (“therapy” or “delivery”)); for “Genome editing”, (“genome editing” and (“therapy” or “delivery”)); for “Gene delivery”, (“gene delivery”); for “Lipid-based”, ((“liposome” or “liposomes” or “lipid nanoparticle” or “lipid nanoparticles”) and (“gene delivery” or “gene therapy”)); for “Polymer-based”, ((“polymer” or “polymers” or “polymeric micelles” or “dendrimers” or “polyion complexes”), and (“gene delivery” or “gene therapy”)).

We summarized the history of gene therapy and non-viral vectors in Figure 1. The development of new delivery systems for nucleic acid and gene medicines can still be expected in the future. To develop the new delivery systems, it is better to understand the in vivo fate of them; then, we summarized it as follows.

## 3. In Vivo Fate

**Figure 2:**
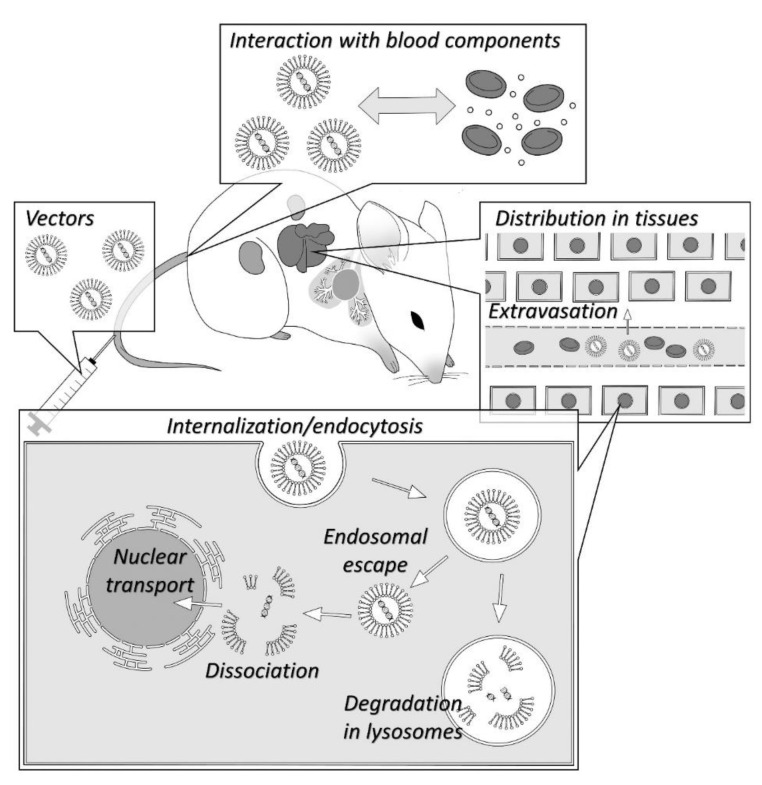
Schematic representation of the in vivo fate of vectors.

Once naked oligonucleotides or gene vectors enter the body, interaction with biological components awaits them [58,59,60]. In particular, the interaction with blood components is a crucial factor in determining the success or failure of in vivo delivery (or gene transfer) after intravenous injection. In addition, distribution to non-target tissues and cells hinders their success. After reaching the target cells, intracellular trafficking is also a hindrance. Since the endosomal/lysosomal pathway degrades the vectors, endosomal escape is necessary for their activity. The double bilayer membrane of the nucleus acts as the final barrier of the cells to defend against the exogenous substances. Each of these processes can act as huge hurdles/barriers for both oligonucleotides and vectors [61]. We summarized the fate of naked oligonucleotides and non-viral vectors after administration (Figure 2).

### 3.1. Interaction with Blood Components

Interaction with blood components is the first step after the administration of the materials into the vasculature [62]. Blood components broadly include blood cells, plasma proteins, and various ions. Since each component can be technically separated, the effect of each component on the interaction with foreign materials can be experimentally analyzed. However, care should be taken to interpret the results. For cationic non-viral vectors such as lipoplexes prepared in nonionic dispersion medium, (example, 5% glucose solution), aggregation and precipitation may occur when mixed with solutions with physiological concentrations of ions (example, saline) [63]. However, the addition of proteins such as serum albumin can inhibit their aggregation [64]. In addition, aggregation and precipitation occur only when red blood cells are suspended in phosphate-buffered saline and mixed with lipoplexes, but adding proteins again to the red blood cell suspension inhibits aggregation [65]. Thus, it is difficult to separately identify the role of each component.

**Figure 3:**
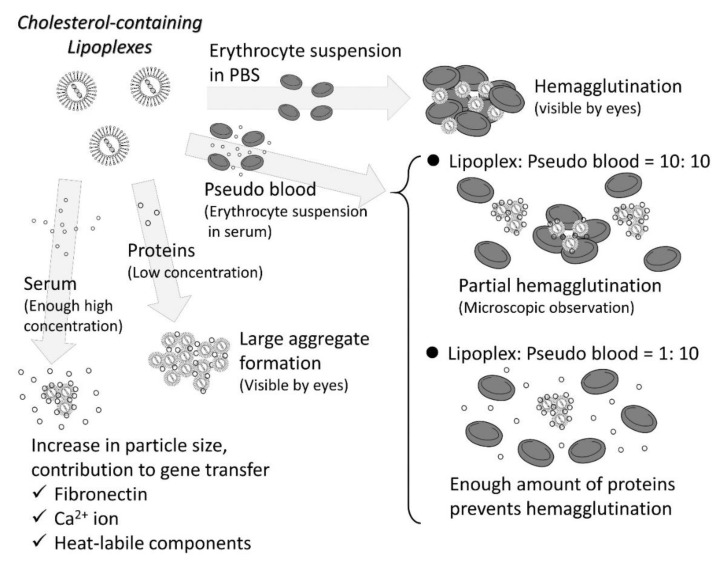
The role of blood components on hemagglutination, formation of large aggregates and size increase of cholesterol-containing lipoplexes, illustrated based on references [71,72].

Pre-incubation of lipoplexes with blood components enables the elucidation of the role of each component (Figure 3). Serum components have been considered as repressors of gene transfer; therefore, serum-free medium has been used for in vitro transfection [66]. The inhibitory effect of serum on transfection can be avoided by increasing the charge ratio, that is, the molar ratio of the positive charge of the cationic liposome to the negative charge of the DNA [67]. If the serum is inactivated by heat treatment in an in vitro experimental system using cultured cells, it is not possible to accurately understand the role of serum in vivo. Sakurai et al. analyzed the role of each component by incubating lipoplexes with erythrocytes or serum prior to administration and showed that gene expression in the lungs was not reduced by preincubation with serum but was reduced by preincubation with erythrocytes [68]. Additionally, in case of intraportal administration of lipoplexes, preincubation with serum increases gene expression in the liver [64]. On the other hand, heat inactivation of the serum reduces gene expression of lipoplexes in the lungs and liver [64]. Therefore, when considering interactions with blood components, blood cells rather than proteins should be considered as inhibitors. Analysis of individual plasma components is also in progress. Lipoproteins are also reported to be major gene expression repressors [69,70]. However, some proteins have been reported to enhance gene expression. How and which components contribute to gene expression is highly dependent on the composition of the vector and conditions of animals. In cationic liposomes composed of the cationic lipid DOTAP and cholesterol, fibronectin significantly contributes to gene expression [71,72]. In hepatitis mice, interaction with albumin significantly contributes to gene expression [73]. Since many plasma proteins adsorb onto the lipoplexes as “protein corona” [74,75,76,77], there may be other important proteins that determine transfection efficiency. It was reported that plasma concentration and the presence of DNA affects the composition of protein corona onto lipoplexes [76,77]. For ionizable lipids that can change their charge with pH and have low surface potential in neutral pH, apolipoprotein E (ApoE) has been reported to make a significant contribution to hepatic gene transfer [78,79]. In addition, the concentration of ApoE affects the delivery efficiency of the cargos. Therefore, when developing non-viral vectors, there is a need to control their interaction with the proteins while suppressing the interaction with blood cells.

Oligonucleotides (ODN) used for antisense and siRNA applications are, in general, single- or double-stranded DNA (or RNA) molecules (typically 5–30 mer), with a range of molecular weight between 2–20 kDa. As this class of drugs is much smaller than plasmid DNA in size, direct injection of naked (unformulated) oligonucleotides has so far been actively pursued. Native DNA and RNA are known to be rapidly eliminated from blood when injected because of poor protein binding and poor metabolic stability [80]. A major strategy to achieve the formulation of injectable naked oligonucleotide drug has been to implement chemical modifications and/or artificial nucleic acids to the body of the oligomers to allow for the utilization of the interaction with serum components and fortify nuclease resistance [81,82,83]. The earliest example is the introduction of a phosphorothioate internucleotide linkage (PS). It has been shown that PS modification greatly enhances albumin binding and tolerability against nuclease digestion, eliciting the prolonged retention in the blood circulation, which gives more opportunity for the drugs to access target tissues [84,85,86]. More recently, Gaus et al. revealed the effect of sequence, sugar modification, and PS content of ASOs on the affinity and selectivity for interacted plasma proteins [87,88]. ASOs with PS modification were shown to associate with more abundant plasma proteins, such as albumin, IgG, transferrin, apolipoprotein A (ApoA), and complement C3 with sub to low micromolar dissociation constants (K_d_), while they bound to less abundant plasma proteins such as histidine-rich glycoprotein (HGS), α-2-macroglobulin (A2M), factor V, and ApoE with 5 to 50 nanomolar K_d_. Interestingly, the flexibility of single-stranded oligonucleotides was found to be important for better plasma protein binding when compared with more rigid counterparts such as PS deoxyadenine (dA) oligomers and ASO/RNA duplexes. At the same time, some knockout mouse studies have provided an important hypothesis that very tight binding with plasma proteins can reduce the activity of ASOs [89].

### 3.2. Distribution to Non-Target Tissues

When using cationic liposomes, gene transfer into the lung is relatively easy [22]. Although an embolic effect is believed to be caused by aggregates produced by the interaction with blood cells, interaction with fibronectin also contributes to pulmonary gene transfer by cationic lipoplexes. This can be explained by the finding that gene expression in the lungs after intravenous administration of lipoplexes is inhibited by 90% due to prior administration of a competitive peptide inhibitor of the interaction of fibronectin and integrins [72]. In any case, if the target tissue is not the lung, transfer to the lung must be inhibited. Other problems include capture by the reticuloendothelial system [90,91]. Therefore, it is necessary to devise ways to promote transfer to the target tissue while increasing blood retention. To improve blood retention to prevent the distribution to non-target tissues, polyethylene glycol (PEG) modification (PEGylation) is the gold standard strategy. PEGylation adds the stealth property to the vector; however, it simultaneously decreases the uptake by target cells. This phenomenon is known as PEG dilemma [92]. There are several strategies to overcome this dilemma, including the use of pH-responsive PEG-lipids [93], enzyme-cleavable PEG-lipids [94,95], and detachable PEG-lipids [96]. Among them, detachable nonionic PEG-lipids, such as PEG-ceramide, 1,2-dimyristoyl-*rac*-glycero-3-methoxypolyethylene glycol (DMG-PEG) and 1,2-dstearoyl-*rac*-glycero-3-methoxypolyethylene glycol (DSG-PEG) are often used for LNP formulation [97,98,99,100]. In general, oligonucleotides are rapidly subjected to renal filtration unless they are heavily chemically modified or encapsulated in a vehicle, such as LNPs. In case of ONPATTRO^®^, an approved siRNA drug, cationic PEG-lipid α-(3-{[1,2-di(myristyloxy)propanoxy]carbonylamino}propyl)-ω-methoxy, polyoxyethylene (PEG-C-DMG) was used for the formulation of the LNP [101]. These PEG-lipids are different from anionic methoxypoly(ethylene glycol)-distearoyl phosphatidylethanolamine (mPEG-DSPE, undetachable [102,103]) in terms of charge, which may be important for detachability from the carriers. In contrast, when it comes to naked oligonucleotides, plasma protein binding is a key factor to be considered to prevent rapid removal by renal filtration, providing a greater chance of being distributed to tissues and cells of interest. These small oligomers require chemical modification to enhance hydrophobicity and charge, based on which oligonucleotide drugs interact with proteins. ASOs with a PS backbone have very high plasma protein binding (more than 85%) [104]. Covalent conjugation of cholesterols, tocopherols, and other fatty acids also help in improving the hydrophobicity and improving the blood retention of the drugs [105,106,107,108,109,110,111,112,113,114]. These lipophilic oligonucleotide drugs are carried by lipoprotein particles, such as high-density lipoprotein (HDL) and low-density lipoprotein (LDL), in addition to plasma proteins, and are directed to a variety of organs, such as the liver, gut, kidney, and steroidogenic organs. However, it should be noted that adding some lipophilicity to the drugs sometimes hinders their activity at target tissues due to increased accumulation of the drugs in non-target tissues [115].

### 3.3. Distribution to Target Tissue

To deliver foreign genes to target tissues, passive and active mechanisms can be involved. As mentioned above, LNPs with ionizable lipids interact with ApoE, which is a subsequence of PEG detachment, as a passive mechanism of delivery. As for the active mechanism, ligand modification (such as sugar galactose and mannose modifications) is useful for delivering the cargo to target cells via specific receptors [38,116,117]. Much effort has been devoted to this receptor-mediated targeting approach for oligonucleotide-based drugs, where a variety of targeting-ligand molecules are directly and covalently conjugated to the drugs. *N*-Acetylgalactosamine (GalNAc) is one of the few successful ligands for both siRNAs [118,119,120,121,122] and ASOs [123,124,125,126,127,128]. The GalNAc ligand can predominantly bind to the asialoglycoprotein receptor (ASGPR), a cell surface lectin receptor, highly and selectively expressed on hepatocytes, eliciting 10- to 60-fold enhancement of the knockdown activity of this class of drugs in the liver. Recently approved Lumisiran (OXLUMO^®^) and Inclisiran (Leqvio^®^) are the first siRNAs that carry GalNAc ligands. Another recent example of ligand-conjugating ASOs was reported by Ämmälä et al., where an ASO conjugated to a peptide ligand that binds to the glucagon-like peptide-1 receptor (GLP1R) was developed to deliver ASOs to pancreatic insulin-secreting β-cells [129]. This conjugate accelerated the uptake of the payload in pancreatic islets and exclusively silenced target gene expression at the intended site.

The intratissue distribution of non-viral vectors that reach the target tissue is also important. If researchers wish to transfer the gene throughout the tissue, the intratissue diffusion must be considered. Particularly in case of gene transfer into cancer cells, diffusion in the cancer tissue (especially with rich interstitial fibers) is a barrier. In addition, if the lesion is confined to a part of the tissue, site-selective gene delivery is desirable.

In most tissues, the vessel wall is a barrier. In other words, gene transfer to cells located outside the blood vessels is extremely difficult. On the other hand, in the liver, spleen, and cancer tissues, the endothelium has fenestrae, which allows gene transfer to cells that lie outside the vessel wall. In case of the liver, the size of the fenestrae present in the sinusoidal endothelial wall is approximately 100 to 160 nm (depending on the species) [130]. Therefore, the size of the carriers should be less than the size of fenestrae to deliver the cargo to hepatocytes. In case of cancer, murine models of cancer cell transplantation can pass through the vessel wall with relative ease, but human solid tumors are often less permeable than tumors in mice, depending on the cancer type [131]. Cabral et al. compared the permeability of polymeric micelles with different sizes (30–100 nm) and found that only micelles measuring 30 nm had a good antitumor effect against poorly permeable pancreatic tumors [132]. The particle size of adeno-associated virus (AAV) is approximately 20 nm [133], and AAV is often used as an efficient viral vector. Therefore, the smaller the particle size, the better is the vessel wall permeability. In this context, much smaller oligonucleotide drugs are being utilized in the naked form as they have the potential to target any tissues by attaching cell-type specific ligand molecules.

### 3.4. Intracellular Trafficking

#### 3.4.1. Uptake Pathway

Cellular uptake of non-viral vectors is usually performed by endocytosis [134,135]. Various endocytic pathways are present, including clathrin-mediated endocytosis, caveolae-mediated endocytosis, flotillin-mediated endocytosis, macropinocytosis, and phagocytosis [136]. In addition, the efficiency of endocytosis is thought to vary depending on the type and density of the receptors, such as LDL receptors and asialoglycoprotein receptors, which have very high capacities [137,138].

Normally, substances taken up by endocytosis into the cell are transferred to the endosome, where they undergo degradation to the lysosome after acidification of the endosome. For this reason, endosomal escape must be considered. Some endocytosis pathways, such as caveolae-mediated endocytosis and macropinocytosis, have been reported to have a different fate after intracellular uptake, and it has been reported that some of the uptake material may not make it to the lysosome [139,140]. At least, the speed of a series of endocytosis is different and the opportunity for escape is ensured because of the long time to transition to lysosomes [136,141,142]. The cellular uptake of naked PS-oligonucleotide-based drugs is thought to be initiated by the adsorption of the drug to the cell surface. This adsorption step is rapid, and a non-energy requiring process [143,144,145]. PS-ASOs’ interactions with membrane surface proteins stimulate multiple cellular pathways to allow the ASOs to trespass into the cytosol. Some of them are called “productive” pathways that elicit pharmacological effect of PS-ASOs, whereas others are called “non-productive” pathways, via which PS-ASOs seem to be directed to late endosomes and lysosomes to accumulate [146,147,148,149]. The productive pathways are thought to include many cell surface receptors that are associated with clathrin-dependent endocytosis, such as G-protein-coupled receptors, scavenger receptors, toll-like receptors, and ASGPR as well as caveolin-dependent surface proteins, such as integrins. The fluid-phase endocytosis and other unknown pathways are involved in the uptake of naked PS-ASOs, among which micropinocytosis is thought to be a non-productive pathway for naked PS-ASOs, while formulated siRNAs apparently utilize this mechanism of uptake.

In contrast, there are intracellular transfer mechanisms that do not depend on endocytosis. For example, Sendai virus (HVJ) releases its contents into the cell by membrane fusion with the plasma membrane, and HVJ-envelope vectors have been developed using this mechanism [150].

#### 3.4.2. Endosomal Escape

We have already stated earlier that non-viral vectors that are taken up by endocytosis need to escape from the endosomes. For this escape, pH-sensitive substances based on endosomal acidification are commonly used [25,79,151,152,153,154]. For naked oligonucleotide drugs, it is still not fully understood how they escape from endosomes. However, some adaptor proteins, such as adaptor-related protein complex 2 subunit mu 1 (AP2M1) and annexin A2, are suggested to play key roles in sorting to direct PS-ASOs (anti-miR) to the productive routes, while vacuolar protein sorting-associated protein 28 homolog (VPS28) and tumor susceptibility gene 101 (TSG101), members of the endosomal sorting complex required for transport-I (ESCRT-I), are associated with the non-productive routes [155].

#### 3.4.3. Subcellular Localization

Cytoskeletons are present in the cytoplasm that restrict the free diffusion of substances [156]. Actin and microtubules are involved in the cytoplasmic transport of non-viral vectors [157]. It has also been reported that DNA undergoes degradation not only in the lysosome, but also in the cytoplasm [158], and thus the stability in the cytoplasm needs to be considered.

In dividing cells, nuclear localization of exogenous DNA is achieved at the nuclear membrane disintegrating time during cell division. In non-dividing cells, the nuclear membrane is intact and mass transport is limited. The molecular weight that can diffuse through the nuclear pores is in the range 60–100 kDa [159], and nuclear localization signals are required to allow larger material to pass through the nuclear pores. Cell lines are proliferative; and therefore, it is necessary to consider cell division when researchers develop non-viral vectors.

Plasmid DNA itself cannot pass through the nuclear pores because its molecular weight is in millions. However, it has been reported that plasmid DNA possessing a specific gene sequence can bind transcription factors and other factors that have nuclear localization signals, and subsequently can pass through the nuclear pores [160,161,162].

A growing body of evidence suggests that each oligonucleotide drug with a different sequence and chemical modification pattern shows a different protein binding profile [163]. This change in protein binding can alter the subcellular localization of the drugs as well as their pharmacological activity and toxicity profiles. To date, approximately 80 intracellular proteins that bind to PS-ASOs have been identified [148,164,165]. Many of the proteins have nucleic acid-binding domains, but others do not. Interaction with some of these proteins, such as 54 kD nuclear RNA-binding protein (P54nrb), positive cofactor 4 (PC4), X-ray repair cross-complementing protein 6 (XRCC6/Ku70), XRCC5/Ku80, and some other miscellaneous proteins with no apparent nucleic acid binding domains such as chaperon proteins heat shock protein 90 (Hsp90) and T-complex protein 1 subunit alpha (Tcp1), membrane-bound annexin A2, tRNA synthase Vars, and cytoskeletal β-actin have been shown to perturb their antisense activity/toxicity profile and subcellular localization. Previous microinjection studies have consistently shown that microinjected oligonucleotide drugs, such as PS-ASOs and locked nucleic acid (LNA)-ASOs, are redistributed from the cytosol to the nucleus within minutes, and only a small amount of ASOs is found in the cytosol, which is presumably ascribed to ASO’s preferential binding to nuclear components during intracellular diffusion [166,167,168].

#### 3.4.4. Dissociation of Genes from Carriers

For effective transcription of mRNA from DNA, DNA should dissociate from its carrier [169]. In normal lipoplexes, this dissociation is less likely to occur, and gene expression is thought to be limited. It has been reported that the transcription and translation efficiency per copy of DNA transferred into the nucleus is 7000 times lower in lipoplexes than in adenoviral vectors [170,171].

#### 3.4.5. Autophagy

Autophagy is considered as an inhibitor of lipofection [172,173]. Inhibition of autophagy by depletion of the autophagy receptor p62 increases transfection efficiency [174]. Chloroquine, an endosomal/autophagosomal acidification inhibitor, is known to increase the transfection efficiency of cationic lipoplexes, while it decreases the efficiency of pH-sensitive liposomes [175,176]. However, it was reported that the autophagy inhibitor vacuolin-1 suppressed the efficacy of siRNA [177]. Thus, the role of autophagy in gene transfer remains controversial.

### 3.5. Metabolism and Excretion

Serum contains nucleases; thus, it is necessary to consider the degradation in blood after intravenous administration of nucleic acid and gene medicines [178,179]. Lysosomes play the role of degradation of nanoparticles in cells [180]. In addition, it was reported that plasmid DNA was instable in cytosol after microinjection [158]. Autophagy might work to degrade nucleic acid and gene medicines taken up by cells. In case of ASOs, urinary excretion should also be considered; then, protein binding is usually required for long-term blood circulation [88].

The chemical-structural unity of the oligonucleotide-based drugs (still a diastereomer mixture if the ones have some stereo-uncontrolled phosphonothioate bonds, though) as well as the growing experience of clinical usage of this class of drugs have helped in investigating their metabolism and excretion characteristics [181]. For example, the 2′-methoxyethyl RNA (MOE)-modified phosphorothioate ASOs, the most clinically-advanced IONIS’s ASO drugs, have consistently been shown to be metabolized primarily by endonucleases, followed by subsequent 3′- and 5′-exonucleases in tissues [182]. Plasma protein binding of the parent MOE ASOs is generally very high (>97%), while these strand-shortened metabolites tend to have much lower affinity to serum components [88]. Eventually, these metabolites and the unbound fraction of the parent ASOs are excreted predominantly from urine, while much less of these drug-related compounds are found in feces [104].

### 3.6. Safety Concern

With viral vectors, there are safety risks, including the production of neutralizing antibodies, hepatitis, and leukemia [183,184,185]. Non-viral vectors are often considered safer than viral vectors, but safety concerns exist for non-viral vectors as well.

For non-viral vectors, cytotoxicity is often an issue in cell culture experiments [40,186,187]. This may be due to the presence of excess cations. There are also reports that cationic liposomes can induce apoptosis [188]. In vivo, innate immunity and hemagglutination, as described below, are more problematic.

Immune responses can occur for non-viral vectors as well as viral vectors. Inflammatory reactions can occur, leading to hepatitis [189]. Acquired immunity to DNA, that is, antibody production, requires little consideration, but attention should be paid to innate immunity. When using plasmid DNA as a vector, the plasmid DNA was amplified by *Escherichia coli* (*E. coli*) and subsequently purified. Since lipopolysaccharide (LPS) from *E. coli* is recognized by Toll-like receptor (TLR) 4 and causes an inflammatory response and inhibits gene expression by the vector, it is necessary to exclude LPS as much as possible [190]. In addition, plasmid DNA differs from mammalian DNA in terms of the methylation pattern of CpG-containing sequences, leading to the recognition by TLR9 and the production of inflammatory cytokines [191]. In the cytoplasm, there are other DNA sensors, that is, the STING/TBK pathway and AIM2/inflammasome, and the production of inflammatory cytokines occurs [192,193]. Immune responses to components other than DNA in non-viral vectors, such as cationic liposomes and cationic polymers, have been reported less frequently and are not well understood. For PEGylated liposomes, the accelerated blood clearance (ABC) phenomenon, in which multiple doses cause the production of anti-PEG IgMs and reduce blood retention [194]. For chitosan, which is a cationic polymer used in gene delivery, anti-chitosan antibodies can also be produced [195]. Thus, especially when using macromolecules, acquired immunity may need to be considered.

When the gene is introduced into a congenitally deficient patient, an immune response to the protein produced by transfection may occur [196]. Immune responses to secreted proteins, and even to gene-expressing cells, occur through the production of antibodies and cellular immunity via major histocompatibility complex class I presentation, which results in the elimination of gene-expressing cells. Conversely, this principle can be applied to DNA vaccines [197]. In DNA vaccines, gene delivery to antigen-presenting cells, such as dendritic cells, acts as a vaccine by eliciting cellular immunity to the protein that is the product of the foreign gene expression. They are thought to be effective against cancer and infectious diseases.

For cationic vectors, capillary embolization is a problem because it can aggregate red blood cells [198]. When erythrocytes are suspended in phosphate-buffered saline, they aggregate at a high rate, but aggregation is inhibited when using a pseudo-blood mixture of erythrocytes and serum and becomes dependent on the mixture ratio of pseudo-blood and cationic vectors [72]. Agglutination is more likely to occur at a mixture ratio of 1:1, which mimics the immediate post-dose period, while aggregation is less likely to occur at a ratio that takes systemic blood volume into account (more than 10:1) [72]. Even at a mixture ratio that is prone to hemagglutination, PEGylation of the vector inhibits it [65].

As with retroviral and lentiviral vectors, random insertion mutagenesis can lead to oncogenesis; for example, the development of leukemia has been reported [199]. For non-viral vectors, the frequency of integration into the host genome is very low when plasmid DNA is used for gene transfer, but random insertion into the genome is reported to occur with highly efficient methods of gene transfer, such as electroporation [200]. This is theoretically avoided in cases of mRNA delivery.

If gene transfer into germ lines such as sperm and eggs occurs, it artificially alters the human race, which presents ethical issues [201].

Safety concerns still exist and have been a major challenge for therapeutic oligonucleotides, even in the post-approval era of this class of drugs. In general, the current forms of ASOs and siRNAs are well tolerated and useful for human treatment; however, substantial side effects, such as inflammation, hepatotoxicity, nephrotoxicity, and thrombocytopenia are occasionally seen in both pre-clinical and clinical settings [202,203]. The production of anti-drug antibody was also pre-clinically and clinically observed for mipomersen, the first FDA-approved systemic antisense drug for hypercholesterolemia. In addition, in some high-affinity 2′-modifications, such as LNA and cEt (constrained 2′-*O*-ethyl), an increased incidence of hepatotoxicity has been identified [204,205,206,207]. Some possible mechanisms have been proposed for this hepatotoxicity: one is a hybridization-dependent off-target pathway, and the other is a sequence-dependent interaction with unwanted proteins. Further investigation is required to elucidate the mechanism of these side effects and broaden the therapeutic index.

## 4. Rational Design

**Figure 4:**
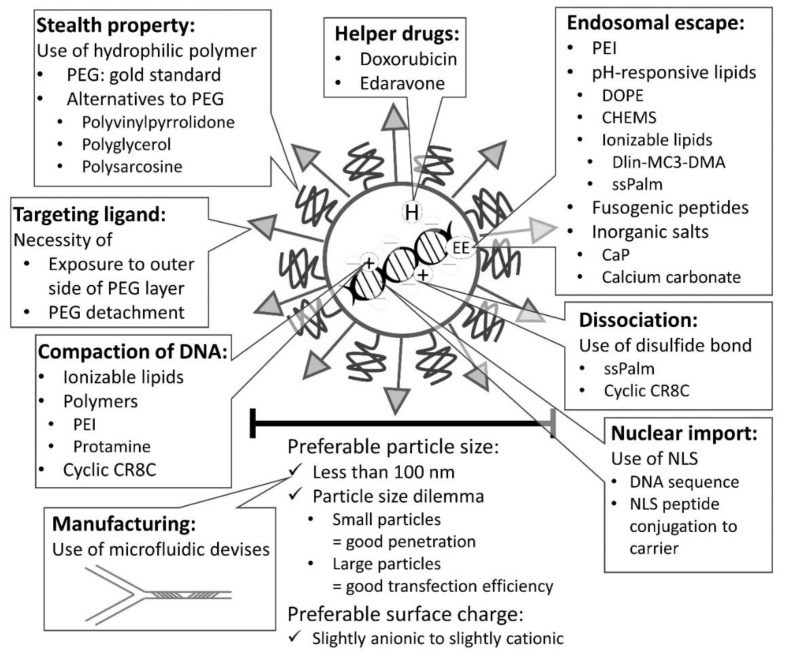
The information for designing ideal non-viral vectors.

**Figure 5:**
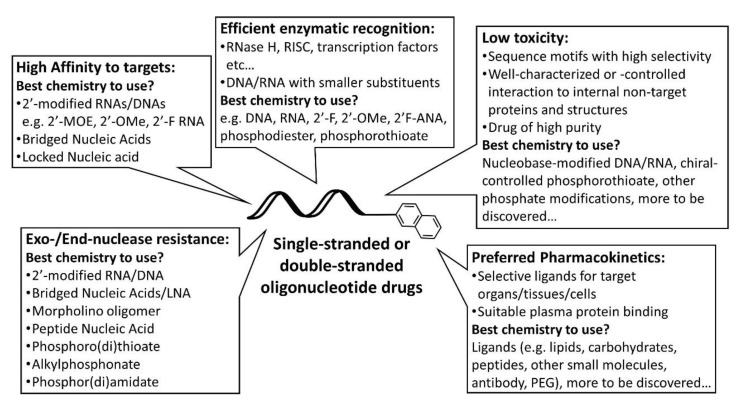
The information for designing ideal oligonucleotide-based drugs.

We illustrated nucleic acid and gene medicines possessing ideal properties for successful delivery (Figure 4 and Figure 5). We summarized the elemental factors to be considered as follows.

### 4.1. Controlling Size of Nanoparticles

The liposome formulation is ahead in terms of the development of a drug delivery system (DDS), and the information can be applied to the development of non-viral vectors, including the solvent, PEGylation, and lyophilization. In terms of particle size, larger particles tend to provide higher gene delivery efficiency of lipoplexes [208], but smaller particles are preferable for controlling disposition in the body.

Non-ionic dispersion medium, such as 5% glucose, can be used to produce small particle lipoplexes [63], but when the lipoplexes are mixed with ionic solvents, the size of the particles increases. Therefore, when used clinically, they should not be mixed with infusions containing electrolytes. Considering the principle of lipoplex formation, it was thought that the cationic liposomes repel each other too strongly to cover the plasmid DNA effectively when the cationic liposomes gather around the plasmid DNA during complex formation in non-ionic dispersion media [63]. The idea was to add an appropriate amount of electrolyte (such as sodium chloride). We hypothesized that the addition of an appropriate amount of electrolyte (e.g., sodium chloride) would suppress the repulsion between cationic liposomes, while ensuring repulsion among lipoplex intermediates. It was found that the addition of 5 to 10 mM sodium chloride to the dispersion medium with 5% glucose stabilized the encapsulation of plasmid DNA, reduced the particle size of lipoplexes, suppressed aggregation when mixed with saline, improved DNA stability in serum, and enhanced pulmonary gene expression after intravenous administration and hepatic gene expression after intraportal administration [63,209]. This strategy using electrolytes for complex formation has been applied to bubble lipopolyplexes, which are composed of anionic liposomes, protamine, plasmid DNA, and echo gas C_3_F_8_, and has shown to improve gas entrapment efficiency [210]. In addition, a moderate concentration of sodium chloride is added to LNP [154]. During plasmid DNA purification, alcohol precipitation is usually performed, but depending on the method, salts are likely to be contaminated. Sodium acetate is less likely to precipitate salts than sodium chloride during alcohol precipitation. Alternatively, it is possible to reduce the amount of salt contamination by mixing a high concentration of plasmid DNA solution in water with ethanol. PEGylation of lipoplexes not only prevents hemagglutination and improves blood retention, but also improves formulation stability. With PEGylation, the particle size of the lipoplexes can be reduced and physical stability can be improved [211]. While preparing lipoplexes, cationic liposomes and plasmid DNA are mixed, but this mixing process can cause variability in the formulation. Here, microfluidic devices can be used to mix the two liquids, and it is expected to produce small nanoparticles in a reproducible manner [212]. A combination of the ethanol injection method is also possible [213], often referred to as lipid nanoparticles (LNPs) rather than liposomes in this case.

### 4.2. Controlling the Interaction with Blood Components

The interaction with blood components defines the distribution of the vector in the body to some extent. Therefore, depending on the target tissue or cell, there may be ways to actively exploit the interaction or prevent it from happening. Interactions with erythrocytes should be prevented in view of the risk of aggregation and embolization. Hemagglutination is thought to be primarily due to electrostatic interactions with the cation. As mentioned earlier, it can be prevented by PEGylation, but it is also useful to make the surface negative potential. To date, liposome/polycation/DNA II, consisting of cationic polymers and anionic liposomes [214], and ternary complexes consisting of cationic polymers and anionic polymers [215] have been developed. However, since there are various components in the blood, some of which are cationic, there is still the possibility of plasma protein binding even when the surface is negatively charged, and the effect of these components on the distribution characteristics of the body must be considered. The blood protein binding ability also matters for therapeutic oligonucleotides to manipulate the pharmacokinetics of smaller drugs. A variety of hydrophobic atoms/groups [110,112,182,216,217], specific binders, such as albumin-avid ibuprofen or proteins, such as albumin [218] have been introduced into therapeutic antisense oligonucleotides. This approach can also help in oligonucleotide drugs being averted from elimination from circulation and directed to tissues, such as the muscle that have not been targeted effectively.

### 4.3. Controlling the Circulation in Vasculatures

PEGs are often used to avoid transfer to non-target tissues, such as capture by the reticuloendothelial system, transfer to the lung due to embolization or interaction with fibronectin, and transfer to the liver due to interaction with ApoE. However, a so-called PEG dilemma exists for PEGylation [92], which also inhibits uptake by target cells and endosomal escape. To circumvent this problem, the use of a PEG lipid, such as PEG-ceramide, which can be detached in the body, is an effective method, as mentioned above. However, it is only buying time to prevent its transfer to tissues, as plasma proteins are thought to interact with it after PEG-ceramide is released in the body. Here, a vector design that detaches the PEG moiety in the target tissue may be effective. For example, a method that utilizes a matrix metalloproteinase expressed in cancer tissue has been reported [93,94,219,220].

ABC phenomenon and presence of anti-PEG antibodies restrict repeated dosage of PEGylated vectors [194,221,222,223]. To evade ABC phenomenon, hydrophilic polymers polyvinylpyrrolidone [224], polyglycerol [225,226], and polysarcosine [227] might be useful as alternatives to PEG.

### 4.4. Selection of Administration Route

The route of vector administration often determines the pharmacokinetics of the vector, and the choice of the route of administration plays an important role in vector development [228]. When administered intravenously, naked plasmid DNA undergoes degradation in the blood and transfers to non-parenchymal cells of the liver, and rarely results in gene expression [229]. However, gene expression is detectable when naked plasmid DNA is injected topically into muscle, heart, liver, and kidney; and especially in muscles, high gene expression can be achieved [230]. Intraarterial and intraportal injection can change the first-pass organ, while the lung is a first-pass organ after intravenous injection. Thus, selection of administration routes is effective in controlling bio-distribution. Oral administration is an attractive route of administration for treatment, but gene delivery is difficult. Even with successful gene transfer, frequent administration is required for epithelial cell turnover. On the other hand, intraperitoneal tissue surface administration allows for efficient gene expression by simply dropping naked plasmid DNA onto the tissue [231]. On the other hand, orally-available ASOs and siRNAs have long been anticipated and just emerged with decent preclinical and clinical data [232,233,234,235]. This advance in this area ascribes to the aforementioned conjugation technology (e.g., GalNAc conjugation) of oligonucleotides and proprietary formulations containing intestinal permeation enhancers. This orally available version of these drugs sees promise in the treatment of chronic diseases as well as alternatives to antibody drugs with only an injection route option. Regarding naked mRNA, the choice of intranodal route (injection to lymph node) is promising for vaccine applications [236]. Transdermal delivery of nucleic acid and gene medicines is usually very difficult since skin has a barrier function. To overcome the barrier, microneedles are a good choice to deliver nucleic acids across the skin [237,238,239]. Thus, selection of administration routes based on the aim of applications is important for the successful delivery of nucleic acid and gene medicines.

### 4.5. Considering Structures of Vessel Wall

The reason for the heterogeneous distribution of non-viral vectors in the tissue after they reach the target tissue is not well understood, but it is thought to be due to heterogeneity in blood flow and expression of receptors. Multiple doses may be helpful. For vascular wall permeation, researchers should pay attention to not only reducing the particle size of the vector, but also to increase the particle size after administration [63]. Again, PEGylation is effective, but when the vascular structure is tight, such as at the blood-brain barrier, vessel wall permeation is difficult to achieve with particle size control. One possibility is to use the transcytotic activity of endothelial cells and use receptors that can cause transcytosis. Such receptors include the transferrin receptor [240].

On the other hand, physical stimuli seem to breach the vessel wall and transfer plasmid DNA into muscle cells when a large volume of plasmid DNA solution is administered into the arteries connected to the muscle [241]. Other methods, such as those using microbubbles or bubble liposomes combined with ultrasound irradiation and tissue suction, are also thought to enable the permeation of the vessel wall [242,243,244].

### 4.6. Targeting Tissues/Cells

In terms of transfer to target cells, it is common to utilize receptors and antigens present in the target cells. For example, sugar modification has been utilized to target liver parenchyma cells with galactose and GalNAc, and non-parenchyma cells and antigen-presenting cells with mannose modification [116,117,118,121,122,123,124,125,245,246,247]. The endocytosis pathway appears to be dependent on non-viral vectors and cell types, with a large contribution of clathrin-mediated endocytosis for lipoplexes and a large contribution of caveolae-mediated endocytosis for PEI complexes [248,249]. Macropinocytosis is an important endocytosis pathway for octaarginine-modified non-viral vectors [250] and uptake of naked plasmid DNA by mesothelial cells on the gastric surface [251]. Antibody- or peptide-oligonucleotide conjugation is another strategy to facilitate targeting delivery of therapeutic oligonucleotides, where the selection of appropriate cell surface receptors, ligands, and chemistry to use for conjugation have been revealed to significantly influence the outcome of the strategy and are not always successful. Additional ligand–receptor combinations effective for the DDS of oligonucleotide drugs have eagerly been anticipated.

Arginine-rich cell penetrating peptides (CPPs) can enter cells via both direct translocation and endocytosis [252]. CPPs are used for the delivery of nucleic acids using chemical conjugation and nanoparticles [253]. Combination of CPPs with targeting ligands is also useful [254].

Some methods exist to open transient pores on the cell membrane by external stimuli. The electroporation method is well known [255,256], but microbubbles and ultrasound irradiation have also been reported to open such pores [257]. Other methods, such as hydrodynamics (the instantaneous administration of a large volume of plasmid DNA solution), are also said to open transient pores [258] as well as rubbing gastric surface [259] and calcium carbonate combination with naked plasmid DNA [260]. These methods are characterized by the fact that they do not require endosomal escape to be considered and very high gene transfer efficiency can be obtained.

It is known that blood vessels in tumor tissues are leaky [261]. It is possible to deliver nanoparticles to tumors passively based on the enhanced permeability and retention (EPR) effect [262,263]. However, the EPR effect seems to be weak in most tumors in humans [131]. It was reported that 30 nm polymeric micelles containing 1,2-diaminocyclohexane-platinum (II) were effective for tumor delivery in a rodent model of human pancreatic cancer. Therefore, small nanoparticles less than 50 nm might be preferable for human cancer therapy. To reduce the size of LNP containing siRNA or mRNA, microfluidic devices are often used [264,265]. However, a size too small seems to be inappropriate for efficacy [264,265]. Reduction in particle size should decrease the amount of cargos per single particle. The contrary effects of reducing size on delivery and efficacy are regarded as “particle size dilemma” [266]. As other issues, tumor microenvironments such as stroma components should be considered [267]. As stromal components, extracellular matrices (ECMs) such as collagen fibers, and high interstitial pressure would restrict the diffusion of nanoparticles in tumor tissues; then, the use of digestive enzymes such as collagenase and hyaluronidase has been investigated to improve nanoparticle delivery and gene transfer efficiency [268,269,270,271]. For active targeting to tumor, several ligands such as arginine-glycine-aspartate (RGD) peptide [272], HER-2-targeting peptide [273] and hyaluronic acid [274] have been used for nanoparticle delivery, and are promising for delivery of nucleic acid and gene medicines [275,276]. Targeting tumor-associated macrophages (TAMs) in the tumor microenvironment is also a useful strategy to combat tumors [277]. It was reported that the combination of mannosylation of bubble lipoplexes and ultrasound irradiation effectively delivered nuclear factor-κB decoy to TAMs, and prolonged survival in mice [278]. A combination of several strategies, including ECM-degradation, active targeting and physical stimuli, would be rational and effective to treat tumors [279,280,281].

### 4.7. Enhancing Endosomal Escape Efficiency

To achieve endosomal escape, it is common to use acidification. Among lipids, phosphatidylethanolamine, such as dioleoyl phosphatidylethanolamine (DOPE), fuses with the endosomal membrane and shows high gene delivery efficiency in cultured cells, but in vivo, it also fuses with erythrocytes, thereby reducing the efficiency [68]. PEI is considered to have a high buffering capacity, and the proton sponge effect causes an influx of counter ions, chloride ions, and endosome acidification, causing the endosome to swell and rupture [282]. There are other ways for endosomal escape using the pH-sensitive fusogenic peptide such as GALA (WEAALAEALAEALAEHLAEALAEALEALAA) [283]. The use of inorganic salts, such as calcium phosphate, can also be helpful. Calcium phosphate and calcium carbonate are expected to cause swelling by increasing the osmotic pressure in the endosomes, owing to their acidification and dissolution. However, it is difficult to control the particle size of inorganic salts. Lipid-calcium phosphate nanoparticles (LCP), with a core composed of calcium phosphate and plasmid DNA, have been developed [38]. However, the manufacturing process is complex and difficult to reproduce.

### 4.8. Considering Intracellular Trafficking

Glutathione is abundant in the cytoplasm and can cleave disulfide bonds. In the LCP, a cyclic CR8C peptide having a disulfide bond was used [38]. Other lipid-like molecules, called ssPalm, are designed to have no charge at physiological pH and become positively charged upon endosomal acidification, thus promoting endosomal escape and cleavage of disulfide bonds in the cytoplasm to dissociate the encapsulated nucleic acids [79,99,154,284].

Some reports suggested that binding nuclear localization signals (NLSs) to dumbbell-shaped DNA promotes nuclear transfer and results in high gene transfer efficiency [285], but this has not been replicated by other groups and no industrial production method for such DNA has been established. Therefore, we believe that the use of cellular transcription factors is a viable option. Attempts to utilize transcription factors that are active only in certain cells and confer cell selectivity have also been reported [286,287]. Since chemical conjugation to DNA might decrease gene expression, NLS-conjugation to carriers of DNA is another option [288,289]. Tammam et al. reported that the moderate density of chemically conjugated NLS peptide (CPKKKRKV) with chitosan via SH-amine bond was effective to deliver the large nanoparticles around 150 nm into the nuclei [288].

### 4.9. Improving Effective Duration

The duration of gene expression by non-viral vectors is generally transient. For example, even with hydrodynamic methods, which allow for highly efficient gene transfer to the liver, gene expression decreases by a factor of 10 for every day after administration [27]. On the other hand, intramuscular administration seems to result in relatively long-term gene expression [26]. The cause of these differences is currently not well understood. Since transcription factor activation, such as NF-κB and AP-1, is involved in gene expression in a variety of ways [290,291,292,293,294], it is likely that the more the gene expression by the transient activation of the transcriptional factors, the faster they decline. In a method that does not rely on such pro-inflammatory events, that is, plasmid DNA that does not contain CpG sequences, there is a prolongation of the gene expression period [295]. Other plasmid DNAs containing the scaffold matrix attachment region (S/MAR) have been developed for long-term gene expression, and combination with CpG-free is effective [244,296,297]. There has also been an attempt to actively utilize integration into the genome (integration) using the PiggyBac transposon and ΦC31 integrase [298,299]. In addition, in vivo genome editing using the CRISPR/Cas9 system delivered by a non-viral vector has been reported [300].

Prolonged gene silencing or knockdown effects are obtained for therapeutic oligonucleotides with a high nuclease resistance and protein binding ability [301]. The effect of the current form of antisense oligonucleotides and siRNA drugs lasts for several months, even with a single injection, and this duration can be further extended by ligand conjugation [302,303]. However, this long durability can also be associated with the above-mentioned side effects. It has been observed that reduction in affinity with undefined proteins by replacing chemistry used in the strand improved the therapeutic index [304]. Therefore, manipulating drug-protein interactions will be a key for this class of drugs with an improved therapeutic index.

### 4.10. Safety Issues

Cytotoxicity and aggregation of erythrocytes can be avoided by modifying PEGs and negative surface potentials. To avoid the proinflammatory cytokines, CpG-free vectors are already available. There are many aspects of safety that are not well understood, and it is important to evaluate various indices, such as hepatitis and renal function.

### 4.11. Helper Drugs

The use of drugs to modulate gene expression has also been reported [266]. The use of doxorubicin is known to enhance foreign gene expression [305,306]. An antioxidant, edaravone, also enhances foreign gene expression [307]. The role of reactive oxygen species (ROS) in gene transfer is currently unclear. In case of hydrodynamics-based transfection, ROS contributes to high gene expression in the liver [308]. Wang et al. reported that there was an appropriate range of edaravone concentration, and significantly high concentration diminished its enhancing effect [307].

## 5. Evaluation Methods

In the development of non-viral vectors, it is necessary to evaluate not only the efficiency of gene transfer, but also the formulation characteristics, internal and cellular behavior, and safety [309]. We summarized methods for evaluation of nucleic acid and gene medicines in Table 1.

### 5.1. Physicochemical Properties

Particle size and zeta potential are important in terms of formulation characteristics for non-viral vectors. The viscosity of the solvent should also be considered as it determines the Brownian motion as well as the particle size. Dynamic light scattering and nano-tracking are often used to determine particle size, and electrophoretic light scattering is often used to determine the zeta potential. For solvent viscosity, a rotational viscometer is suitable if the solvent volume is available. An agarose gel electrophoresis retardation assay was used to assess the formation of the complexes. For external accessibility of encapsulated DNA, ethidium bromide intercalation assay is used. Scanning electron microscopy and transmission electron microscopy are often used to evaluate vector structures. Other structural characterizations are also performed by small-angle X-ray scattering.

### 5.2. Reporter Genes

Luciferases, such as firefly, are often used for quantitative evaluation of gene expression. The luminescence reaction by luciferase was measured using a luminometer, and the sensitivity was very high. Although in vivo imaging in live mice is also possible, the detection of luminescence produced from luciferase and luciferin is inefficient because the wavelength is not within the range of the biological window. It is possible to use a variety of substrates, such as Akalumine-HCl, which emit near-infrared light [310]. Firefly luciferase is unstable in vivo and is suitable for monitoring gene expression over time. This means that if researchers use a stable reporter gene; they will be able to measure the amount of gene expression product rather than evaluating the gene expression itself. Firefly luciferase, on the other hand, seems to be unsuitable as a secreted type from the perspective of blood retention. Instead, *Gaussia* luciferase has better blood retention and is easier to detect as a secreted type. Secreted alkaline phosphatase is used as a reporter gene of the secreted type.

Fluorescent proteins are used to identify and detect gene expression-positive cells. ZsGreen1 is recommended as a green fluorescent protein because of its brightness. Among red fluorescent proteins, tdTomato has high fluorescence, and 488 nm laser excitation makes it suitable for use in flow cytometers, but not for multicolor observation in confocal laser microscopy [311]. Typically, fluorescent proteins are observed either by isolating cells, such as by collagenase perfusion, or by observing tissue sections. Because of the loss of spatial information when observing tissue sections, the authors have focused on the recently developed technique of tissue optical clearing and have succeeded in visualizing gene expression introduced by the hydrodynamic method and the spatial distribution of administered plasmid DNA in tissue by combining tissue transparency and confocal laser microscopy [311,312].

### 5.3. Biodistribution

Radiolabeling is the least troublesome method for the quantification of biodistribution. For example, plasmid DNA ^32^P labeling and ^111^In labeling have been used [117,313]. Although non-RI quantification is also desirable, it is difficult to quantify with fluorescence due to autofluorescence of biological components, long-wavelength fluorescence probes such as Cy7 may be able to follow the internal dynamics. In terms of tissue distribution, there is a method to observe live mice using a confocal laser microscope, but it is difficult because of the requirement of high fluorescent dye density [314]. This problem can be solved to some extent with the use of multiphoton microscopy [315].

In situ tissue perfusion system such as liver perfusion is useful to analyze local disposition characteristics of drugs [316,317]. Tissue perfusion enables evaluation of the role of interaction with blood component in local disposition characteristics [64]. Analyses based on perfusion of other tissues such as brain [318], lung [319], kidney [320], muscle [321] and tumor [322] are also possible, but the technics are relatively difficult compared with liver perfusion.

### 5.4. Subcellular Localization

For tracking the subcellular distribution of non-viral vectors, fluorescence is suitable. For cultured cells, monolayer culture is less troublesome and allows for quantitative analysis and evaluation of subcellular distribution along with visualization of endosomes, lysosomes, microtubules, and nuclei [323]. For three-dimensional cultures, such as spheroids, quantitative analysis is difficult because fluorescence is harder to reach inside the culture. For vectors administered to living organisms, it is possible to evaluate their subcellular distribution by observing tissue sections or analyzing them three-dimensionally using the tissue clearing described earlier, but even in this case, quantitative analysis is difficult.

The size of nanoparticles used in non-viral vectors is smaller than the diffraction limit of visible light, and accurate analysis of their intracellular behavior has traditionally been limited to electron microscopy. However, the recent development of super-resolution microscopy that exceeds the diffraction limit of visible light [324]; such technological innovations are likely to provide new insights into the intracellular behavior of non-viral vectors.

Endosomal escape efficiency is one of determinant factors of successful nucleic acid and gene delivery; thus, quantitation of endosomal escape is an important issue. Recently, Jiang et al. reported the quantitation method [325]. They have synthesized a molecular probe based on deglycosylation-dependent *renilla* luciferase. The probe is a kind of prodrug, and activatable in cytosol. Subsequently, they revealed the importance of endosomal escape, not uptake, by screening polymer library to transfect mRNA. Such evaluation methodologies can promote the development of effective nucleic acid and gene medicines.

Predicting in vivo efficacy and the safety of oligonucleotide drugs based on in vitro screening has still been a major hurdle mainly due to the fact that the precise in vivo fate of naked oligonucleotide drugs has not been fully elucidated; using a conventional in vitro lipofection often causes false positives because it facilitates the entirely different uptake mechanisms from the ones the naked drugs utilize in vivo [326]. A few alternative in vitro transfection systems have so far been devised: (1) using an engineered murine primary hepatocyte cell line [146], (2) gymnosis (=free-uptake) [327], (3) Ca^2+^-enrichment in medium (CEM) transfection [328]. The gymnosis and CEM are both very powerful and easy-to-access methodologies because they work on a variety of cell lines and oligonucleotides, which are performed simply by adding oligonucleotides of interest in a culture medium. While the gymnotic method requires higher concentrations of the drugs (~ µM) and longer waiting time (more than three days) under standard cell culture conditions before analysis, the CEM requires a higher concentration of Ca^2+^ (~9 mM) in a standard culture medium to help form albumin aggregates, which stimulate the uptake and decrease the effective concentration of oligonucleotides (nM to µM) as well as the waiting time (1 to 3 days). The CEM method has been shown to exhibit better prediction of in vivo activity of ASOs without cytotoxicity; suggesting that the CEM transfection method is useful not only for a high-throughput screening of therapeutic oligonucleotides, but also to further study the cellular uptake mechanisms and intracellular trafficking of naked oligonucleotides.

### 5.5. Toxicity

The quantification of inflammatory cytokines is essential to understand the safety of non-viral vectors. There is also a high need to evaluate hepatitis, renal function, and other factors.

## 6. Prospects and Conclusions

Since elemental technologies have been developed, their combination is effective to design ideal delivery systems (Figure 4 and Figure 5). For nucleic acid delivery, effective systems have been developed as described above. We believe that the major unsolved problem in non-viral gene delivery is heterogeneity. Taking the differences between mRNA and DNA deliveries into consideration, the biggest issue in DNA delivery should be nuclear import. Incorporation of NLSs into the LNP systems might be a useful strategy.

Targeting to the liver seems to be relatively easy, but the control of delivered cell type(s) is required ideally. For targeting tumor, the regulation in size and utilization of targeting ligand(s) are useful strategies. However, delivery to all cells in tumor (100% of cells) is almost impossible. Therefore, the combination of nucleic acid and gene medicines with immune checkpoint inhibitors is promising to treat tumor. There are other methods of delivery to other tissues such as the brain, but the development of technology that can target a wider range of cell types is expected. For targeting other tissues, the major problem is the structure of blood vessels. Small molecules can be delivered to other tissues such as the kidney and bones [329,330]. However, gene delivery to these tissues should be more difficult. A combination with physical stimuli is an attractive option to target other tissues such as the kidney, peritoneum, heart, and muscle [244,297,331,332,333,334].

About duration, efficacy of stable ASOs are long-lasting (several months) [335,336]. Duration of LNP containing siRNA or mRNA is relatively short (several days to weeks) [337,338]; thus, a more frequent dosage than chemically modified stable ASOs is required for LNP. In addition, a transient expression from mRNA-LNP would be suitable to vaccine application [339]. The duration of most non-viral gene delivery systems is yet shorter (several weeks) [340,341] than that of adeno-associated viral vectors (several months to years) [342,343]. However, the presence of neutralizing antibodies against adeno-associated viral vectors [344] clearly indicates the necessity of the development of non-viral vectors.

The toxicity issues should restrict the clinical use of non-viral vectors. The dilemma of toxicity and effectiveness of transfection is known, and several attempts have been made to resolve the dilemma [345,346]. A possible solution for the toxicity issues is local administration. Another option is with the use of helper drugs such as edaravone to decrease toxicity [307].

The tissue environment differs depending on the disease state. For example, loss of fenestrae on sinusoidal endothelium should be considered for liver targeting in fibrotic condition [347,348]. Since ONPATTRO uses ApoE for liver delivery [349], it might be ineffective in ApoE deficient patients [350]. It was reported that kidney conditions affected the disposition of drugs [351,352]. In fact, uremic toxin indoxyl sulfate activates macrophages [353,354]. In addition, the role of albumin interaction with lipoplexes is different in healthy and hepatitis mice [73], and sonoporation effectively transfers plasmid DNA in peritoneal fibrotic site, while linear PEI fails to transfer [355]. These indicate the necessity of considering disease state for delivery of nucleic acid and gene medicines.

Adding too many functions to delivery systems complicates the formulation of them; therefore, it is important to develop simple production methods. Peng et al. have developed the one-step preparation method of lipid-coated multiple drug-containing calcium carbonate nanoparticles based on ethanol injection [272,356]. Such simple preparation methods are expected for GMP-grade production of nucleic acid and gene medicines.

In conclusion, the development of nucleic acid/oligonucleotide and gene delivery systems is a cycle of rational design of vectors based on known information, evaluation of their formulation characteristics and intra- and intracellular behavior, and further improvement. Therefore, it is necessary to establish evaluation methods as well as to develop the vectors themselves. There are many issues such as transfer efficiency, duration, safety, and ethics. We hope that this review will provide useful information for the development of delivery systems.

## Figures and Tables

**Table 1:** Evaluation methods for nucleic acid and gene medicines.

Processes/Properties	Methods	Note
Physicochemical properties		
Particle size	Dynamic light scattering	Most popular Necessity of solvent viscosity and refractive index
	Nano tracking analysis	Determination of the particle concentration: possible
ζ potential	Laser doppler electrophoresis	Necessity of solvent viscosity, refractive index and dielectric constant
Morphology	Electron microscopy	Cryo EM: preferable
Complexation/Encapsulation	Agarose gel retardation assay	Solution composition: to be considered
	EtBr intercalation	Relatively low sensitivity
	Picogreen/Ribogreen assay	Determination of encapsulation efficiency: possible
In vivo fate		
Interaction with blood components	Microscopy (phase contrast/ differential interference contrast)	Aggregation/hemagglutination
	Proteomics (LC-MS/MS)	Technique: required
Blood/tissue concentration	Radioisotopes	High sensitivity Controlled area: necessary
	Fluorescent labeling	Non-RI Multiplex: possible
Local disposition	Tissue perfusion system	Technique: required
Spatial distribution in tissues	Fluorescent labeling/fluorescent protein, tissue clearing, confocal/ multi-photon/super-resolution microscopy	Multiplex: possible Relationship with biological events and states: analyzable
Efficacy of oligonucleotide drugs	Ca^2+^ enrichment in medium	Prediction of in vivo efficacy by in vitro experiments
	Gymnosis	Prediction of in vivo efficacy by in vitro experiments
Gene expression		
Reporter genes	Luciferase (Firefly, *Renilla*, *Gaussia*, Synthetic), luminometer	Cost-effective, high sensitivity
	Fluorescent protein Fluorescent/confocal microscopy	Determination of positive cells and spatial distribution
Therapeutic genes	Realtime PCR	More direct than reporter gene assay Procedure: cumbersome
Cellular trafficking		
Association/uptake	Fluorescent labeling	Separating association and uptake: possible, but difficult
Endosome/lysosome/cytosolic localization	Fluorescent probes (Lysotracker etc.) Confocal/super-resolution microscopy	Resolution: important Quantitation: possible, but difficult
Dissociation	Different color labeling of nucleic acids/genes and carriers Confocal/super-resolution microscopy	Quantitation: possible, but difficult
Autophagy	Fluorescent probes (DAL green etc.) or anti-LC3 antibody Confocal/super-resolution microscopy	Quantitation: possible, but difficult
Nuclear localization	Fluorescent labeling, nuclear stain (DAPI etc.) Confocal/super-resolution microscopy	Quantitation: possible, but difficult
Toxicity		
Inflammation	ELISA (serum cytokines) Plate reader	Kits: available
Hepatitis	Serum AST and ALT, photometer	Kits: available
Renal function	Serum creatinine test, photometer	Kits: available
Others	Thin tissue section Hematoxylin and eosin staining	Procedure: cumbersome

## Data Availability

Data is contained within the article.

## References

[1] Gonzaga-Jauregui C., Lupski J.R., Gibbs R.A. (2012). Human Genome Sequencing in Health and Disease. Annu. Rev. Med..

[2] Sarantis P., Koustas E., Papadimitropoulou A., Papavassiliou A.G., Karamouzis M.V. (2020). Pancreatic ductal adenocarcinoma: Treatment hurdles, tumor microenvironment and immunotherapy. World J. Gastrointest. Oncol..

[3] Vonderheide R.H. (2020). CD40 Agonist Antibodies in Cancer Immunotherapy. Annu. Rev. Med..

[4] Yaghoubi S., Karimi M.H., Lotfinia M., Gharibi T., Mahi-Birjand M., Kavi E., Hosseini F., Sineh Sepehr K., Khatami M., Bagheri N. (2020). Potential drugs used in the antibody-drug conjugate (ADC) architecture for cancer therapy. J. Cell. Physiol..

[5] Crooke S.T., Witztum J.L., Bennett C.F., Baker B.F. (2018). RNA-Targeted Therapeutics. Cell Metab..

[6] Lau Y.-T.K., Ramaiyer M., Johnson D.E., Grandis J.R. (2019). Targeting STAT3 in Cancer with Nucleotide Therapeutics. Cancers.

[7] Hammond S.M. (2015). An overview of microRNAs. Adv. Drug Deliv. Rev..

[8] Kulabhusan P.K., Hussain B., Yüce M. (2020). Current Perspectives on Aptamers as Diagnostic Tools and Therapeutic Agents. Pharmaceutics.

[9] Fang L., Du W.W., Lyu J., Dong J., Zhang C., Yang W., He A., Kwok Y.S.S., Ma J., Wu N. (2018). Enhanced breast cancer progression by mutant p53 is inhibited by the circular RNA circ-Ccnb1. Cell Death Differ..

[10] Xu Y., Qiu A., Peng F., Tan X., Wang J., Gong X. (2020). Exosomal transfer of circular RNA FBXW7 ameliorates the chemoresistance to oxaliplatin in colorectal cancer by sponging miR-18b-5p. Neoplasma.

[11] Li C., Samulski R.J. (2020). Engineering adeno-associated virus vectors for gene therapy. Nat. Rev. Genet..

[12] Ginn S.L., Amaya A.K., Alexander I.E., Edelstein M., Abedi M.R. (2018). Gene therapy clinical trials worldwide to 2017: An update. J. Gene Med..

[13] Yi Y., Hahm S.H., Lee K.H. (2005). Retroviral gene therapy: Safety issues and possible solutions. Curr. Gene Ther..

[14] Shirley J.L., de Jong Y.P., Terhorst C., Herzog R.W. (2020). Immune Responses to Viral Gene Therapy Vectors. Mol. Ther..

[15] McCutchan J.H., Pagano J.S. (1968). Enchancement of the infectivity of simian virus 40 deoxyribonucleic acid with diethylaminoethyl-dextran. J. Natl. Cancer Inst..

[16] Johnson S.M., Bangham A.D. (1969). Potassium permeability of single compartment liposomes with and without valinomycin. Biochim. Biophys. Acta.

[17] Johnson S.M., Bangham A.D., Hill M.W., Korn E.D. (1971). Single bilayer liposomes. Biochim. Biophys. Acta.

[18] Bangham A.D. (1972). Lipid bilayers and biomembranes. Annu. Rev. Biochem..

[19] Mannino R.J., Allebach E.S., Strohl W.A. (1979). Encapsulation of high molecular weight DNA in large unilamellar phospholipid vesicles. Dependence on the size of the DNA. FEBS Lett..

[20] Dimitriadis G.J. (1979). Entrapment of plasmid DNA in liposomes. Nucleic Acids Res..

[21] Felgner P.L., Gadek T.R., Holm M., Roman R., Chan H.W., Wenz M., Northrop J.P., Ringold G.M., Danielsen M. (1987). Lipofection: A highly efficient, lipid-mediated DNA-transfection procedure. Proc. Natl. Acad. Sci. USA.

[22] Song Y.K., Liu F., Chu S., Liu D. (1997). Characterization of cationic liposome-mediated gene transfer in vivo by intravenous administration. Hum. Gene Ther..

[23] Semple S.C., Klimuk S.K., Harasym T.O., Dos Santos N., Ansell S.M., Wong K.F., Maurer N., Stark H., Cullis P.R., Hope M.J. (2001). Efficient encapsulation of antisense oligonucleotides in lipid vesicles using ionizable aminolipids: Formation of novel small multilamellar vesicle structures. Biochim. Biophys. Acta.

[24] Semple S.C., Akinc A., Chen J., Sandhu A.P., Mui B.L., Cho C.K., Sah D.W., Stebbing D., Crosley E.J., Yaworski E. (2010). Rational design of cationic lipids for siRNA delivery. Nat. Biotechnol..

[25] Kulkarni J.A., Myhre J.L., Chen S., Tam Y.Y.C., Danescu A., Richman J.M., Cullis P.R. (2017). Design of lipid nanoparticles for in vitro and in vivo delivery of plasmid DNA. Nanomedicine.

[26] Wolff J.A., Malone R.W., Williams P., Chong W., Acsadi G., Jani A., Felgner P.L. (1990). Direct gene transfer into mouse muscle in vivo. Science.

[27] Liu F., Song Y., Liu D. (1999). Hydrodynamics-based transfection in animals by systemic administration of plasmid DNA. Gene Ther..

[28] Zhang G., Budker V., Wolff J.A. (1999). High levels of foreign gene expression in hepatocytes after tail vein injections of naked plasmid DNA. Hum. Gene Ther..

[29] Neumann E., Schaefer-Ridder M., Wang Y., Hofschneider P.H. (1982). Gene transfer into mouse lyoma cells by electroporation in high electric fields. EMBO J..

[30] Reiss M., Jastreboff M.M., Bertino J.R., Narayanan R. (1986). DNA-mediated gene transfer into epidermal cells using electroporation. Biochem. Biophys. Res. Commun..

[31] Wells J.M., Li L.H., Sen A., Jahreis G.P., Hui S.W. (2000). Electroporation-enhanced gene delivery in mammary tumors. Gene Ther..

[32] Sakai M., Nishikawa M., Thanaketpaisarn O., Yamashita F., Hashida M. (2005). Hepatocyte-targeted gene transfer by combination of vascularly delivered plasmid DNA and in vivo electroporation. Gene Ther..

[33] Endoh M., Koibuchi N., Sato M., Morishita R., Kanzaki T., Murata Y., Kaneda Y. (2002). Fetal gene transfer by intrauterine injection with microbubble-enhanced ultrasound. Mol. Ther..

[34] Suzuki R., Takizawa T., Negishi Y., Hagisawa K., Tanaka K., Sawamura K., Utoguchi N., Nishioka T., Maruyama K. (2007). Gene delivery by combination of novel liposomal bubbles with perfluoropropane and ultrasound. J. Control. Release.

[35] Liu F., Huang L. (2002). Noninvasive gene delivery to the liver by mechanical massage. Hepatology.

[36] Mukai H., Kawakami S., Hashida M. (2008). Renal press-mediated transfection method for plasmid DNA and siRNA to the kidney. Biochem. Biophys. Res. Commun..

[37] Shimizu K., Kawakami S., Hayashi K., Kinoshita H., Kuwahara K., Nakao K., Hashida M., Konishi S. (2012). In vivo site-specific transfection of naked plasmid DNA and siRNAs in mice by using a tissue suction device. PLoS ONE.

[38] Hu Y., Haynes M.T., Wang Y., Liu F., Huang L. (2013). A highly efficient synthetic vector: Nonhydrodynamic delivery of DNA to hepatocyte nuclei in vivo. ACS Nano.

[39] Goula D., Remy J.S., Erbacher P., Wasowicz M., Levi G., Abdallah B., Demeneix B.A. (1998). Size, diffusibility and transfection performance of linear PEI/DNA complexes in the mouse central nervous system. Gene Ther..

[40] Morimoto K., Nishikawa M., Kawakami S., Nakano T., Hattori Y., Fumoto S., Yamashita F., Hashida M. (2003). Molecular weight-dependent gene transfection activity of unmodified and galactosylated polyethyleneimine on hepatoma cells and mouse liver. Mol. Ther..

[41] Choi J.S., Nam K., Park J.Y., Kim J.B., Lee J.K., Park J.S. (2004). Enhanced transfection efficiency of PAMAM dendrimer by surface modification with L-arginine. J. Control. Release.

[42] Uchida S., Kataoka K. (2019). Design concepts of polyplex micelles for in vivo therapeutic delivery of plasmid DNA and messenger RNA. J. Biomed. Mater. Res. A.

[43] Naito M., Chaya H., Toh K., Kim B.S., Hayashi K., Fukushima S., Nagata T., Yokota T., Kataoka K., Miyata K. (2021). Structural tuning of oligonucleotides for enhanced blood circulation properties of unit polyion complexes prepared from two-branched poly(ethylene glycol)-block-poly(l-lysine). J. Control. Release.

[44] Saw P.E., Song E.W. (2020). siRNA therapeutics: A clinical reality. Sci. China Life Sci..

[45] Hu B., Zhong L., Weng Y., Peng L., Huang Y., Zhao Y., Liangcd X.-J. (2020). Therapeutic siRNA: State of the art. Signal Transduct. Target. Ther..

[46] Samaridou E., Heyes J., Lutwyche P. (2020). Lipid nanoparticles for nucleic acid delivery: Current perspectives. Adv. Drug Deliv. Rev..

[47] Nicola M., Alsafi Z., Sohrabi C., Kerwan A., Al-Jabir A., Iosifidis C., Agha M., Agha R. (2020). The socio-economic implications of the coronavirus pandemic (COVID-19): A review. Int. J. Surg..

[48] Oliver S.E., Gargano J.W., Marin M., Wallace M., Curran K.G., Chamberland M., McClung N., Campos-Outcalt D., Morgan R.L., Mbaeyi S. (2021). The Advisory Committee on Immunization Practices’ Interim Recommendation for Use of Moderna COVID-19 Vaccine—United States, December 2020. MMWR. Morb. Mortal. Wkly. Rep..

[49] Oliver S.E., Gargano J.W., Marin M., Wallace M., Curran K.G., Chamberland M., McClung N., Campos-Outcalt D., Morgan R.L., Mbaeyi S. (2020). The Advisory Committee on Immunization Practices’ Interim Recommendation for Use of Pfizer-BioNTech COVID-19 Vaccine—United States, December 2020. MMWR. Morb. Mortal. Wkly. Rep..

[50] Liu M.A. (2019). A Comparison of Plasmid DNA and mRNA as Vaccine Technologies. Vaccines.

[51] De Jong W., Leal L., Buyze J., Pannus P., Guardo A.C., Salgado M., Mothe B., Moltó J., Moron-Lopez S., Gálvez C. (2019). Therapeutic Vaccine in Chronically HIV-1-Infected Patients: A Randomized, Double-Blind, Placebo-Controlled Phase IIa Trial with HTI-TriMix. Vaccines.

[52] Meleshko A.N., Petrovskaya N.A., Savelyeva N., Vashkevich K.P., Doronina S.N., Sachivko N.V. (2017). Phase I clinical trial of idiotypic DNA vaccine administered as a complex with polyethylenimine to patients with B-cell lymphoma. Hum. Vaccines Immunother..

[53] Kim H., Kim E.H., Kwak G., Chi S.-G., Kim S.H., Yang Y. (2020). Exosomes: Cell-Derived Nanoplatforms for the Delivery of Cancer Therapeutics. Int. J. Mol. Sci..

[54] Muhammad S.A. (2021). Are extracellular vesicles new hope in clinical drug delivery for neurological disorders?. Neurochem. Int..

[55] Ortega A., Martinez-Arroyo O., Forner M.J., Cortes R. (2020). Exosomes as Drug Delivery Systems: Endogenous Nanovehicles for Treatment of Systemic Lupus Erythematosus. Pharmaceutics.

[56] Lara P., Chan A., Cruz L.J., Quest A.G., Kogan M.J. (2020). Exploiting the Natural Properties of Extracellular Vesicles in Targeted Delivery towards Specific Cells and Tissues. Pharmaceutics.

[57] Yamayoshi A., Oyama S., Kishimoto Y., Konishi R., Yamamoto T., Kobori A., Harada H., Ashihara E., Sugiyama H., Murakami A. (2020). Development of Antibody–Oligonucleotide Complexes for Targeting Exosomal MicroRNA. Pharmaceutics.

[58] Johannes L., Lucchino M. (2018). Current Challenges in Delivery and Cytosolic Translocation of Therapeutic RNAs. Nucleic Acid Ther..

[59] Ogris M., Wagner E. (2002). Tumor-targeted gene transfer with DNA polyplexes. Somat. Cell Mol. Genet..

[60] Alp G., Aydogan N. (2020). Lipid-based mucus penetrating nanoparticles and their biophysical interactions with pulmonary mucus layer. Eur. J. Pharm. Biopharm..

[61] Nishikawa M., Huang L. (2001). Nonviral vectors in the new millennium: Delivery barriers in gene transfer. Hum. Gene Ther..

[62] Karmali P.P., Simberg D. (2011). Interactions of nanoparticles with plasma proteins: Implication on clearance and toxicity of drug delivery systems. Expert Opin. Drug Deliv..

[63] Fumoto S., Kawakami S., Ito Y., Shigeta K., Yamashita F., Hashida M. (2004). Enhanced hepatocyte-selective in vivo gene expression by stabilized galactosylated liposome/plasmid DNA complex using sodium chloride for complex formation. Mol. Ther..

[64] Fumoto S., Kawakami S., Shigeta K., Higuchi Y., Yamashita F., Hashida M. (2005). Interaction with blood components plays a crucial role in asialoglycoprotein receptor-mediated in vivo gene transfer by galactosylated lipoplex. J. Pharmacol. Exp. Ther..

[65] Eliyahu H., Servel N., Domb A.J., Barenholz Y. (2002). Lipoplex-induced hemagglutination: Potential involvement in intravenous gene delivery. Gene Ther..

[66] Gao X., Huang L. (1991). A novel cationic liposome reagent for efficient transfection of mammalian cells. Biochem. Biophys. Res. Commun..

[67] Yang J.P., Huang L. (1997). Overcoming the inhibitory effect of serum on lipofection by increasing the charge ratio of cationic liposome to DNA. Gene Ther..

[68] Sakurai F., Nishioka T., Saito H., Baba T., Okuda A., Matsumoto O., Taga T., Yamashita F., Takakura Y., Hashida M. (2001). Interaction between DNA-cationic liposome complexes and erythrocytes is an important factor in systemic gene transfer via the intravenous route in mice: The role of the neutral helper lipid. Gene Ther..

[69] Tandia B.M., Vandenbranden M., Wattiez R., Lakhdar Z., Ruysschaert J.M., Elouahabi A. (2003). Identification of human plasma proteins that bind to cationic lipid/DNA complex and analysis of their effects on transfection efficiency: Implications for intravenous gene transfer. Mol. Ther..

[70] Tandia B.M., Lonez C., Vandenbranden M., Ruysschaert J.M., Elouahabi A. (2005). Lipid mixing between lipoplexes and plasma lipoproteins is a major barrier for intravenous transfection mediated by cationic lipids. J. Biol. Chem..

[71] Yoshikawa N., Sakamoto K., Mizuno S., Sakaguchi J., Miyamoto H., Mine T., Sasaki H., Fumoto S., Nishida K. (2011). Multiple components in serum contribute to hepatic transgene expression by lipoplex in mice. J. Gene Med..

[72] Yoshikawa N., Fumoto S., Nakashima M., Shimokawa K., Miyamoto H., Nishida K. (2013). The role of fibronectin in pulmonary gene transfer following intravenous administration of lipoplex in mice. Biol. Pharm. Bull..

[73] Yoshikawa N., Fumoto S., Yoshikawa K., Hu D., Okami K., Kato R., Nakashima M., Miyamoto H., Nishida K. (2020). Interaction of Lipoplex with Albumin Enhances Gene Expression in Hepatitis Mice. Pharmaceutics.

[74] Motta S., Rondelli V., Cantu’ L., Del Favero E., Aureli M., Pozzi D., Caracciolo G., Brocca P. (2016). What the cell surface does not see: The gene vector under the protein corona. Colloids Surf. B Biointerfaces.

[75] Capriotti A.L., Caracciolo G., Caruso G., Foglia P., Pozzi D., Samperi R., Laganà A. (2011). Differential analysis of “protein corona” profile adsorbed onto different nonviral gene delivery systems. Anal. Biochem..

[76] Caracciolo G., Pozzi D., Capriotti A.L., Cavaliere C., Foglia P., Amenitsch H., Laganà A. (2011). Evolution of the Protein Corona of Lipid Gene Vectors as a Function of Plasma Concentration. Langmuir.

[77] Capriotti A.L., Caracciolo G., Caruso G., Foglia P., Pozzi D., Samperi R., Laganà A. (2011). DNA affects the composition of lipoplex protein corona: A proteomics approach. Proteomics.

[78] Akinc A., Querbes W., De S., Qin J., Frank-Kamenetsky M., Jayaprakash K.N., Jayaraman M., Rajeev K.G., Cantley W.L., Dorkin J.R. (2010). Targeted delivery of RNAi therapeutics with endogenous and exogenous ligand-based mechanisms. Mol. Ther..

[79] Akita H., Noguchi Y., Hatakeyama H., Sato Y., Tange K., Nakai Y., Harashima H. (2015). Molecular Tuning of a Vitamin E-Scaffold pH-Sensitive and Reductive Cleavable Lipid-like Material for Accelerated In Vivo Hepatic siRNA Delivery. ACS Biomater. Sci. Eng..

[80] Dias N., Stein C.A. (2002). Antisense oligonucleotides: Basic concepts and mechanisms. Mol. Cancer Ther..

[81] Yamamoto T., Nakatani M., Narukawa K., Obika S. (2011). Antisense drug discovery and development. Future Med. Chem..

[82] Khvorova A., Watts J.K. (2017). The chemical evolution of oligonucleotide therapies of clinical utility. Nat. Biotechnol..

[83] Wan W.B., Seth P.P. (2016). The Medicinal Chemistry of Therapeutic Oligonucleotides. J. Med. Chem..

[84] Crooke S.T., Vickers T., Lima W.F., Wu H., Crooke S.T. (2007). Mechanisms of antisense drug action, an introduction. Antisense Drug Technology: Principles, Strategies, and Applications.

[85] Crooke S.T. (2017). Molecular Mechanisms of Antisense Oligonucleotides. Nucleic Acid Ther..

[86] Crooke S.T., Seth P.P., Vickers T.A., Liang X.H. (2020). The Interaction of Phosphorothioate-Containing RNA Targeted Drugs with Proteins Is a Critical Determinant of the Therapeutic Effects of These Agents. J. Am. Chem. Soc..

[87] Gaus H.J., Gupta R., Chappell A.E., Østergaard M.E., Swayze E.E., Seth P.P. (2019). Characterization of the interactions of chemically-modified therapeutic nucleic acids with plasma proteins using a fluorescence polarization assay. Nucleic Acids Res..

[88] Yu R.Z., Kim T.W., Hong A., Watanabe T.A., Gaus H.J., Geary R.S. (2007). Cross-species pharmacokinetic comparison from mouse to man of a second-generation antisense oligonucleotide, ISIS 301012, targeting human apolipoprotein B-100. Drug Metab. Dispos..

[89] Shemesh C.S., Yu R.Z., Gaus H.J., Seth P.P., Swayze E.E., Bennett F.C., Geary R.S., Henry S.P., Wang Y. (2016). Pharmacokinetic and Pharmacodynamic Investigations of ION-353382, a Model Antisense Oligonucleotide: Using Alpha-2-Macroglobulin and Murinoglobulin Double-Knockout Mice. Nucleic Acid Ther..

[90] Matsumura Y. (2014). The Drug Discovery by NanoMedicine and its Clinical Experience. Jpn. J. Clin. Oncol..

[91] Charbe N.B., Amnerkar N.D., Ramesh B., Tambuwala M.M., Bakshi H.A., Aljabali A.A.A., Khadse S.C., Satheeshkumar R., Satija S., Metha M. (2020). Small interfering RNA for cancer treatment: Overcoming hurdles in delivery. Acta Pharm. Sin. B.

[92] Hatakeyama H., Akita H., Harashima H. (2013). The polyethyleneglycol dilemma: Advantage and disadvantage of PEGylation of liposomes for systemic genes and nucleic acids delivery to tumors. Biol. Pharm. Bull..

[93] Shin J., Shum P., Thompson D.H. (2003). Acid-triggered release via dePEGylation of DOPE liposomes containing acid-labile vinyl ether PEG-lipids. J. Control. Release.

[94] Terada T., Iwai M., Kawakami S., Yamashita F., Hashida M. (2006). Novel PEG-matrix metalloproteinase-2 cleavable peptide-lipid containing galactosylated liposomes for hepatocellular carcinoma-selective targeting. J. Control. Release.

[95] Hatakeyama H., Akita H., Kogure K., Oishi M., Nagasaki Y., Kihira Y., Ueno M., Kobayashi H., Kikuchi H., Harashima H. (2007). Development of a novel systemic gene delivery system for cancer therapy with a tumor-specific cleavable PEG-lipid. Gene Ther..

[96] Shi F., Wasungu L., Nomden A., Stuart M.C., Polushkin E., Engberts J.B., Hoekstra D. (2002). Interference of poly(ethylene glycol)-lipid analogues with cationic-lipid-mediated delivery of oligonucleotides; role of lipid exchangeability and non-lamellar transitions. Biochem. J..

[97] Monck M.A., Mori A., Lee D., Tam P., Wheeler J.J., Cullis P.R., Scherrer P. (2000). Stabilized plasmid-lipid particles: Pharmacokinetics and plasmid delivery to distal tumors following intravenous injection. J. Drug Target..

[98] Ambegia E., Ansell S., Cullis P., Heyes J., Palmer L., MacLachlan I. (2005). Stabilized plasmid-lipid particles containing PEG-diacylglycerols exhibit extended circulation lifetimes and tumor selective gene expression. Biochim. Biophys. Acta.

[99] Akita H., Ishiba R., Togashi R., Tange K., Nakai Y., Hatakeyama H., Harashima H. (2015). A neutral lipid envelope-type nanoparticle composed of a pH-activated and vitamin E-scaffold lipid-like material as a platform for a gene carrier targeting renal cell carcinoma. J. Control. Release.

[100] Zhu X., Tao W., Liu D., Wu J., Guo Z., Ji X., Bharwani Z., Zhao L., Zhao X., Farokhzad O.C. (2017). Surface De-PEGylation Controls Nanoparticle-Mediated siRNA Delivery In Vitro and In Vivo. Theranostics.

[101] Zhang X., Goel V., Attarwala H., Sweetser M.T., Clausen V.A., Robbie G.J. (2020). Patisiran Pharmacokinetics, Pharmacodynamics, and Exposure-Response Analyses in the Phase 3 APOLLO Trial in Patients with Hereditary Transthyretin-Mediated (hATTR) Amyloidosis. J. Clin. Pharmacol..

[102] Webb M.S., Saxon D., Wong F.M., Lim H.J., Wang Z., Bally M.B., Choi L.S., Cullis P.R., Mayer L.D. (1998). Comparison of different hydrophobic anchors conjugated to poly(ethylene glycol): Effects on the pharmacokinetics of liposomal vincristine. Biochim. Biophys. Acta.

[103] Lechanteur A., Furst T., Evrard B., Delvenne P., Hubert P., Piel G. (2016). PEGylation of lipoplexes: The right balance between cytotoxicity and siRNA effectiveness. Eur. J. Pharm. Sci..

[104] Geary R.S., Norris D., Yu R., Bennett C.F. (2015). Pharmacokinetics, biodistribution and cell uptake of antisense oligonucleotides. Adv. Drug Deliv. Rev..

[105] Wolfrum C., Shi S., Jayaprakash K.N., Jayaraman M., Wang G., Pandey R.K., Rajeev K.G., Nakayama T., Charrise K., Ndungo E.M. (2007). Mechanisms and optimization of in vivo delivery of lipophilic siRNAs. Nat. Biotechnol..

[106] Nishina T., Numata J., Nishina K., Yoshida-Tanaka K., Nitta K., Piao W., Iwata R., Ito S., Kuwahara H., Wada T. (2015). Chimeric Antisense Oligonucleotide Conjugated to α-Tocopherol. Mol. Ther. Nucleic Acids.

[107] Nishina K., Piao W., Yoshida-Tanaka K., Sujino Y., Nishina T., Yamamoto T., Nitta K., Yoshioka K., Kuwahara H., Yasuhara H. (2015). DNA/RNA heteroduplex oligonucleotide for highly efficient gene silencing. Nat. Commun..

[108] Nishina K., Unno T., Uno Y., Kubodera T., Kanouchi T., Mizusawa H., Yokota T. (2008). Efficient in vivo delivery of siRNA to the liver by conjugation of alpha-tocopherol. Mol. Ther..

[109] Soutschek J., Akinc A., Bramlage B., Charisse K., Constien R., Donoghue M., Elbashir S., Geick A., Hadwiger P., Harborth J. (2004). Therapeutic silencing of an endogenous gene by systemic administration of modified siRNAs. Nature.

[110] Hvam M.L., Cai Y., Dagnæs-Hansen F., Nielsen J.S., Wengel J., Kjems J., Howard K.A. (2017). Fatty Acid-Modified Gapmer Antisense Oligonucleotide and Serum Albumin Constructs for Pharmacokinetic Modulation. Mol. Ther..

[111] Nikan M., Osborn M.F., Coles A.H., Godinho B.M., Hall L.M., Haraszti R.A., Hassler M.R., Echeverria D., Aronin N., Khvorova A. (2016). Docosahexaenoic Acid Conjugation Enhances Distribution and Safety of siRNA upon Local Administration in Mouse Brain. Mol. Ther. Nucleic Acids.

[112] Prakash T.P., Mullick A.E., Lee R.G., Yu J., Yeh S.T., Low A., Chappell A.E., Østergaard M.E., Murray S., Gaus H.J. (2019). Fatty acid conjugation enhances potency of antisense oligonucleotides in muscle. Nucleic Acids Res..

[113] Wada S., Obika S., Shibata M.A., Yamamoto T., Nakatani M., Yamaoka T., Torigoe H., Harada-Shiba M. (2012). Development of a 2′,4′-BNA/LNA-based siRNA for Dyslipidemia and Assessment of the Effects of Its Chemical Modifications In Vivo. Mol. Ther. Nucleic Acids.

[114] Wada S., Yasuhara H., Wada F., Sawamura M., Waki R., Yamamoto T., Harada-Shiba M., Obika S. (2016). Evaluation of the effects of chemically different linkers on hepatic accumulations, cell tropism and gene silencing ability of cholesterol-conjugated antisense oligonucleotides. J. Control. Release.

[115] Wada F., Yamamoto T., Ueda T., Sawamura M., Wada S., Harada-Shiba M., Obika S. (2018). Cholesterol-GalNAc Dual Conjugation Strategy for Reducing Renal Distribution of Antisense Oligonucleotides. Nucleic Acid Ther..

[116] Kawakami S., Sato A., Nishikawa M., Yamashita F., Hashida M. (2000). Mannose receptor-mediated gene transfer into macrophages using novel mannosylated cationic liposomes. Gene Ther..

[117] Kawakami S., Fumoto S., Nishikawa M., Yamashita F., Hashida M. (2000). In vivo gene delivery to the liver using novel galactosylated cationic liposomes. Pharm. Res..

[118] Nair J.K., Willoughby J.L., Chan A., Charisse K., Alam M.R., Wang Q., Hoekstra M., Kandasamy P., Kel’in A.V., Milstein S. (2014). Multivalent N-acetylgalactosamine-conjugated siRNA localizes in hepatocytes and elicits robust RNAi-mediated gene silencing. J. Am. Chem. Soc..

[119] Weingärtner A., Bethge L., Weiss L., Sternberger M., Lindholm M.W. (2020). Less Is More: Novel Hepatocyte-Targeted siRNA Conjugates for Treatment of Liver-Related Disorders. Mol. Ther. Nucleic Acids.

[120] Matsuda S., Keiser K., Nair J.K., Charisse K., Manoharan R.M., Kretschmer P., Peng C.G., VKel’in A., Kandasamy P., Willoughby J.L. (2015). siRNA conjugates carrying sequentially assembled trivalent N-acetylgalactosamine linked through nucleosides elicit robust gene silencing in vivo in hepatocytes. ACS Chem. Biol..

[121] Rajeev K.G., Nair J.K., Jayaraman M., Charisse K., Taneja N., O’Shea J., Willoughby J.L., Yucius K., Nguyen T., Shulga-Morskaya S. (2015). Hepatocyte-specific delivery of siRNAs conjugated to novel non-nucleosidic trivalent N-acetylgalactosamine elicits robust gene silencing in vivo. Chembiochem.

[122] Sharma V.K., Osborn M.F., Hassler M.R., Echeverria D., Ly S., Ulashchik E.A., Martynenko-Makaev Y.V., Shmanai V.V., Zatsepin T.S., Khvorova A. (2018). Novel Cluster and Monomer-Based GalNAc Structures Induce Effective Uptake of siRNAs in Vitro and in Vivo. Bioconjug. Chem..

[123] Prakash T.P., Graham M.J., Yu J., Carty R., Low A., Chappell A., Schmidt K., Zhao C., Aghajan M., Murray H.F. (2014). Targeted delivery of antisense oligonucleotides to hepatocytes using triantennary N-acetyl galactosamine improves potency 10-fold in mice. Nucleic Acids Res..

[124] Østergaard M.E., Yu J., Kinberger G.A., Wan W.B., Migawa M.T., Vasquez G., Schmidt K., Gaus H.J., Murray H.M., Low A. (2015). Efficient Synthesis and Biological Evaluation of 5’-GalNAc Conjugated Antisense Oligonucleotides. Bioconjug. Chem..

[125] Yamamoto T., Sawamura M., Wada F., Harada-Shiba M., Obika S. (2016). Serial incorporation of a monovalent GalNAc phosphoramidite unit into hepatocyte-targeting antisense oligonucleotides. Bioorg. Med. Chem..

[126] Shemesh C.S., Yu R.Z., Gaus H.J., Greenlee S., Post N., Schmidt K., Migawa M.T., Seth P.P., Zanardi T.A., Prakash T.P. (2016). Elucidation of the Biotransformation Pathways of a Galnac3-conjugated Antisense Oligonucleotide in Rats and Monkeys. Mol. Ther. Nucleic Acids.

[127] Kinberger G.A., Prakash T.P., Yu J., Vasquez G., Low A., Chappell A., Schmidt K., Murray H.M., Gaus H., Swayze E.E. (2016). Conjugation of mono and di-GalNAc sugars enhances the potency of antisense oligonucleotides via ASGR mediated delivery to hepatocytes. Bioorg. Med. Chem. Lett..

[128] Watanabe A., Nakajima M., Kasuya T., Onishi R., Kitade N., Mayumi K., Ikehara T., Kugimiya A. (2016). Comparative Characterization of Hepatic Distribution and mRNA Reduction of Antisense Oligonucleotides Conjugated with Triantennary N-Acetyl Galactosamine and Lipophilic Ligands Targeting Apolipoprotein B. J. Pharmacol. Exp. Ther..

[129] Ämmälä C., Drury W.J., Knerr L., Ahlstedt I., Stillemark-Billton P., Wennberg-Huldt C., Andersson E.M., Valeur E., Jansson-Löfmark R., Janzén D. (2018). Targeted delivery of antisense oligonucleotides to pancreatic β-cells. Sci. Adv..

[130] Wisse E., Jacobs F., Topal B., Frederik P., De Geest B. (2008). The size of endothelial fenestrae in human liver sinusoids: Implications for hepatocyte-directed gene transfer. Gene Ther..

[131] Danhier F. (2016). To exploit the tumor microenvironment: Since the EPR effect fails in the clinic, what is the future of nanomedicine?. J. Control. Release.

[132] Cabral H., Matsumoto Y., Mizuno K., Chen Q., Murakami M., Kimura M., Terada Y., Kano M.R., Miyazono K., Uesaka M. (2011). Accumulation of sub-100 nm polymeric micelles in poorly permeable tumours depends on size. Nat. Nanotechnol..

[133] Yates V.J., el-Mishad A.M., McCormick K.J., Trentin J.J. (1973). Isolation and characterization of an Avian adenovirus-associated virus. Infect. Immun..

[134] Pichon C., Billiet L., Midoux P. (2010). Chemical vectors for gene delivery: Uptake and intracellular trafficking. Curr. Opin. Biotechnol..

[135] Islam M.A., Firdous J., Choi Y.-J., Yun C.-H., Cho C.-S. (2014). Regulation of endocytosis by non-viral vectors for efficient gene activity. J. Biomed. Nanotechnol..

[136] El-Sayed A., Harashima H. (2013). Endocytosis of gene delivery vectors: From clathrin-dependent to lipid raft-mediated endocytosis. Mol. Ther..

[137] Spady D.K. (1992). Hepatic clearance of plasma low density lipoproteins. Semin. Liver Dis..

[138] Hu J., Liu J., Yang D., Lu M., Yin J. (2014). Physiological roles of asialoglycoprotein receptors (ASGPRs) variants and recent advances in hepatic-targeted delivery of therapeutic molecules via ASGPRs. Protein Pept. Lett..

[139] Pelkmans L., Kartenbeck J., Helenius A. (2001). Caveolar endocytosis of simian virus 40 reveals a new two-step vesicular-transport pathway to the ER. Nat. Cell. Biol..

[140] Pelkmans L., Helenius A. (2003). Insider information: What viruses tell us about endocytosis. Curr. Opin. Cell Biol..

[141] Henley J.R., Krueger E.W., Oswald B.J., McNiven M.A. (1998). Dynamin-mediated internalization of caveolae. J. Cell Biol..

[142] Damm E.M., Pelkmans L., Kartenbeck J., Mezzacasa A., Kurzchalia T., Helenius A. (2005). Clathrin- and caveolin-1-independent endocytosis: Entry of simian virus 40 into cells devoid of caveolae. J. Cell Biol..

[143] Ugarte-Uribe B., Pérez-Rentero S., Lucas R., Aviñó A., Reina J.J., Alkorta I., Eritja R., Morales J.C. (2010). Synthesis, cell-surface binding, and cellular uptake of fluorescently labeled glucose-DNA conjugates with different carbohydrate presentation. Bioconjug. Chem..

[144] Cheng C.J., Saltzman W.M. (2011). Enhanced siRNA delivery into cells by exploiting the synergy between targeting ligands and cell-penetrating peptides. Biomaterials.

[145] Yu C., Brussaard A.B., Yang X., Listerud M., Role L.W. (1993). Uptake of antisense oligonucleotides and functional block of acetylcholine receptor subunit gene expression in primary embryonic neurons. Dev. Genet..

[146] Koller E., Vincent T.M., Chappell A., De S., Manoharan M., Bennett C.F. (2011). Mechanisms of single-stranded phosphorothioate modified antisense oligonucleotide accumulation in hepatocytes. Nucleic Acids Res..

[147] Crooke S.T., Wang S., Vickers T.A., Shen W., Liang X.H. (2017). Cellular uptake and trafficking of antisense oligonucleotides. Nat. Biotechnol..

[148] Liang X.H., Sun H., Shen W., Crooke S.T. (2015). Identification and characterization of intracellular proteins that bind oligonucleotides with phosphorothioate linkages. Nucleic Acids Res..

[149] Tanowitz M., Hettrick L., Revenko A., Kinberger G.A., Prakash T.P., Seth P.P. (2017). Asialoglycoprotein receptor 1 mediates productive uptake of N-acetylgalactosamine-conjugated and unconjugated phosphorothioate antisense oligonucleotides into liver hepatocytes. Nucleic Acids Res..

[150] Kaneda Y., Nakajima T., Nishikawa T., Yamamoto S., Ikegami H., Suzuki N., Nakamura H., Morishita R., Kotani H. (2002). Hemagglutinating virus of Japan (HVJ) envelope vector as a versatile gene delivery system. Mol. Ther..

[151] Farhood H., Serbina N., Huang L. (1995). The role of dioleoyl phosphatidylethanolamine in cationic liposome mediated gene transfer. Biochim. Biophys. Acta.

[152] Guo X., Gagne L., Chen H., Szoka F.C. (2014). Novel ortho ester-based, pH-sensitive cationic lipid for gene delivery in vitro and in vivo. J. Liposome Res..

[153] Khalil I.A., Kimura S., Sato Y., Harashima H. (2018). Synergism between a cell penetrating peptide and a pH-sensitive cationic lipid in efficient gene delivery based on double-coated nanoparticles. J. Control. Release.

[154] Tanaka H., Takahashi T., Konishi M., Takata N., Gomi M., Shirane D., Miyama R., Hagiwara S., Yamasaki Y., Sakurai Y. (2020). Self-Degradable Lipid-Like Materials Based on “Hydrolysis accelerated by the intra-Particle Enrichment of Reactant (HyPER)” for Messenger RNA Delivery. Adv. Funct. Mater..

[155] Wagenaar T.R., Tolstykh T., Shi C., Jiang L., Zhang J., Li Z., Yu Q., Qu H., Sun F., Cao H. (2015). Identification of the endosomal sorting complex required for transport-I (ESCRT-I) as an important modulator of anti-miR uptake by cancer cells. Nucleic Acids Res..

[156] Döhner K., Sodeik B. (2005). The Role of the Cytoskeleton during Viral Infection. Curr. Top. Microbiol. Immunol..

[157] Ondrej V., Lukásová E., Falk M., Kozubek S. (2007). The role of actin and microtubule networks in plasmid DNA intracellular trafficking. Acta Biochim. Pol..

[158] Lechardeur D., Sohn K.J., Haardt M., Joshi P.B., Monck M., Graham R.W., Beatty B., Squire J., O’Brodovich H., Lukacs G.L. (1999). Metabolic instability of plasmid DNA in the cytosol: A potential barrier to gene transfer. Gene Ther..

[159] Wang R., Brattain M.G. (2007). The maximal size of protein to diffuse through the nuclear pore is larger than 60kDa. FEBS Lett..

[160] Dean D.A. (1997). Import of plasmid DNA into the nucleus is sequence specific. Exp Cell Res..

[161] Dean D.A., Dean B.S., Muller S., Smith L.C. (1999). Sequence requirements for plasmid nuclear import. Exp. Cell Res..

[162] Bai H., Lester G.M.S., Petishnok L.C., Dean D.A. (2017). Cytoplasmic transport and nuclear import of plasmid DNA. Biosci. Rep..

[163] Crooke S.T., Vickers T.A., Liang X.H. (2020). Phosphorothioate modified oligonucleotide-protein interactions. Nucleic Acids Res..

[164] Weidner D.A., Valdez B.C., Henning D., Greenberg S., Busch H. (1995). Phosphorothioate oligonucleotides bind in a non sequence-specific manner to the nucleolar protein C23/nucleolin. FEBS Lett..

[165] Abdul-Manan N., Williams K.R. (1996). hnRNP A1 binds promiscuously to oligoribonucleotides: Utilization of random and homo-oligonucleotides to discriminate sequence from base-specific binding. Nucleic Acids Res..

[166] Buntz A., Killian T., Schmid D., Seul H., Brinkmann U., Ravn J., Lindholm M., Knoetgen H., Haucke V., Mundigl O. (2019). Quantitative fluorescence imaging determines the absolute number of locked nucleic acid oligonucleotides needed for suppression of target gene expression. Nucleic Acids Res..

[167] Leonetti J.P., Mechti N., Degols G., Gagnor C., Lebleu B. (1991). Intracellular distribution of microinjected antisense oligonucleotides. Proc. Natl. Acad. Sci. USA.

[168] Fisher T.L., Terhorst T., Cao X., Wagner R.W. (1993). Intracellular disposition and metabolism of fluorescently-labeled unmodified and modified oligonucleotides microinjected into mammalian cells. Nucleic Acids Res..

[169] Zhang W., Kang X., Yuan B., Wang H., Zhang T., Shi M., Zheng Z., Zhang Y., Peng C., Fan X. (2019). Nano-Structural Effects on Gene Transfection: Large, Botryoid-Shaped Nanoparticles Enhance DNA Delivery via Macropinocytosis and Effective Dissociation. Theranostics.

[170] Hama S., Akita H., Ito R., Mizuguchi H., Hayakawa T., Harashima H. (2006). Quantitative comparison of intracellular trafficking and nuclear transcription between adenoviral and lipoplex systems. Mol. Ther..

[171] Hama S., Akita H., Iida S., Mizuguchi H., Harashima H. (2007). Quantitative and mechanism-based investigation of post-nuclear delivery events between adenovirus and lipoplex. Nucleic Acids Res..

[172] Roberts R., Al-Jamal W.T., Whelband M., Thomas P., Jefferson M., van den Bossche J., Powell P.P., Kostarelos K., Wileman T. (2013). Autophagy and formation of tubulovesicular autophagosomes provide a barrier against nonviral gene delivery. Autophagy.

[173] Thomas O.S., Weber W. (2019). Overcoming Physiological Barriers to Nanoparticle Delivery-Are We There Yet?. Front. Bioeng. Biotechnol..

[174] Tsuchiya M., Ogawa H., Koujin T., Kobayashi S., Mori C., Hiraoka Y., Haraguchi T. (2016). Depletion of autophagy receptor p62/SQSTM1 enhances the efficiency of gene delivery in mammalian cells. FEBS Lett..

[175] Legendre J.Y., Szoka F.C. (1992). Delivery of plasmid DNA into mammalian cell lines using pH-sensitive liposomes: Comparison with cationic liposomes. Pharm. Res..

[176] Maitani Y., Igarashi S., Sato M., Hattori Y. (2007). Cationic liposome (DC-Chol/DOPE=1:2) and a modified ethanol injection method to prepare liposomes, increased gene expression. Int. J. Pharm..

[177] Li T., Yue J., Huang L., Yang M. (2019). Autophagy inhibitor Vacuolin-1 interferes with lipid-based small interference RNA delivery. Biochem. Biophys. Res. Commun..

[178] Geary R.S., Leeds J.M., Fitchett J., Burckin T., Truong L., Spainhour C., Creek M., Levin A.A. (1997). Pharmacokinetics and metabolism in mice of a phosphorothioate oligonucleotide antisense inhibitor of C-raf-1 kinase expression. Drug Metab. Dispos..

[179] Wu J., Chen J., Feng Y., Tian H., Chen X. (2019). Tumor microenvironment as the “regulator” and “target” for gene therapy. J. Gene Med..

[180] Liu C.-G., Han Y.-H., Kankala R.K., Wang S.-B., Chen A.-Z. (2020). Subcellular Performance of Nanoparticles in Cancer Therapy. Int. J. Nanomed..

[181] Post N., Yu R., Greenlee S., Gaus H., Hurh E., Matson J., Wang Y. (2019). Metabolism and Disposition of Volanesorsen, a 2′-O-(2 methoxyethyl) Antisense Oligonucleotide, Across Species. Drug Metab. Dispos..

[182] Geary R.S., Yu R.Z., Watanabe T., Henry S.P., Hardee G.E., Chappell A., Matson J., Sasmor H., Cummins L., Levin A.A. (2003). Pharmacokinetics of a Tumor Necrosis Factor—A Phosphorothioate 2′-O-(2-Methoxyethyl) Modified Antisense Oligonucleotide: Comparison across Species. Drug Metab. Dispos..

[183] Nidetz N.F., McGee M.C., Tse L.V., Li C., Cong L., Li Y., Huang W. (2020). Adeno-associated viral vector-mediated immune responses: Understanding barriers to gene delivery. Pharmacol. Ther..

[184] Sakurai F., Nakamura S.-I., Akitomo K., Shibata H., Terao K., Kawabata K., Hayakawa T., Mizuguchi H. (2008). Transduction Properties of Adenovirus Serotype 35 Vectors After Intravenous Administration into Nonhuman Primates. Mol. Ther..

[185] Modlich U., Schambach A., Brugman M.H., Wicke D.C., Knoess S., Li Z., Maetzig T., Rudolph C., Schlegelberger B., Baum C. (2008). Leukemia induction after a single retroviral vector insertion in Evi1 or Prdm16. Leukemia.

[186] Sakurai F., Inoue R., Nishino Y., Okuda A., Matsumoto O., Taga T., Yamashita F., Takakura Y., Hashida M. (2000). Effect of DNA/liposome mixing ratio on the physicochemical characteristics, cellular uptake and intracellular trafficking of plasmid DNA/cationic liposome complexes and subsequent gene expression. J. Control. Release.

[187] Fischer D., Bieber T., Li Y., Elsässer H.P., Kissel T. (1999). A novel non-viral vector for DNA delivery based on low molecular weight, branched polyethylenimine: Effect of molecular weight on transfection efficiency and cytotoxicity. Pharm. Res..

[188] Aramaki Y., Takano S., Tsuchiya S. (1999). Induction of apoptosis in macrophages by cationic liposomes. FEBS Lett..

[189] Ito Y., Kawakami S., Charoensit P., Higuchi Y., Hashida M. (2009). Evaluation of proinflammatory cytokine production and liver injury induced by plasmid DNA/cationic liposome complexes with various mixing ratios in mice. Eur. J. Pharm. Biopharm..

[190] Butash K.A., Natarajan P., Young A., Fox D.K. (2000). Reexamination of the effect of endotoxin on cell proliferation and transfection efficiency. Biotechniques.

[191] Yasuda K., Ogawa Y., Yamane I., Nishikawa M., Takakura Y. (2005). Macrophage activation by a DNA/cationic liposome complex requires endosomal acidification and TLR9-dependent and -independent pathways. J. Leukoc. Biol..

[192] Ishikawa H., Ma Z., Barber G.N. (2009). STING regulates intracellular DNA-mediated, type I interferon-dependent innate immunity. Nature.

[193] Man S.M., Karki R., Kanneganti T.D. (2016). AIM2 inflammasome in infection, cancer, and autoimmunity: Role in DNA sensing, inflammation, and innate immunity. Eur. J. Immunol..

[194] Lila A.S.A., Ishida T. (2019). Anti-PEG IgM Production via a PEGylated Nanocarrier System for Nucleic Acid Delivery. Methods Mol. Biol..

[195] Sorlier P., Hartmann D.J., Denuzière A., Viton C., Domard A. (2003). Preparation and development of anti-chitosan antibodies. J. Biomed. Mater. Res. A.

[196] Le Guiner C., Stieger K., Snyder R.O., Rolling F., Moullier P. (2007). Immune responses to gene product of inducible promoters. Curr. Gene Ther..

[197] Li L., Petrovsky N. (2016). Molecular mechanisms for enhanced DNA vaccine immunogenicity. Expert Rev. Vaccines.

[198] Litzinger D.C., Brown J.M., Wala I., Kaufman S.A., Van G.Y., Farrell C.L., Collins D. (1996). Fate of cationic liposomes and their complex with oligonucleotide in vivo. Biochim. Biophys. Acta.

[199] Hacein-Bey-Abina S., Von Kalle C., Schmidt M., McCormack M.P., Wulffraat N., Leboulch P., Lim A., Osborne C.S., Pawliuk R., Morillon E. (2003). LMO2-associated clonal T cell proliferation in two patients after gene therapy for SCID-X1. Science.

[200] Wang Z., Troilo P.J., Wang X., Griffiths T.G., Pacchione S.J., Barnum A.B., Harper L.B., Pauley C.J., Niu Z., Denisova L. (2004). Detection of integration of plasmid DNA into host genomic DNA following intramuscular injection and electroporation. Gene Ther..

[201] Smith K.R. (2003). Gene therapy: Theoretical and bioethical concepts. Arch. Med. Res..

[202] Frazier K.S. (2015). Antisense oligonucleotide therapies: The promise and the challenges from a toxicologic pathologist’s perspective. Toxicol. Pathol..

[203] Wittrup A., Lieberman J. (2015). Knocking down disease: A progress report on siRNA therapeutics. Nat. Rev. Genet..

[204] Swayze E.E., Siwkowski A.M., Wancewicz E.V., Migawa M.T., Wyrzykiewicz T.K., Hung G., Monia B.P., Bennett C.F. (2007). Antisense oligonucleotides containing locked nucleic acid improve potency but cause significant hepatotoxicity in animals. Nucleic Acids Res..

[205] Burdick A.D., Sciabola S., Mantena S.R., Hollingshead B.D., Stanton R., Warneke J.A., Zeng M., Martsen E., Medvedev A., Makarov S.S. (2014). Sequence motifs associated with hepatotoxicity of locked nucleic acid--modified antisense oligonucleotides. Nucleic Acids Res..

[206] Dieckmann A., Hagedorn P.H., Burki Y., Brügmann C., Berrera M., Ebeling M., Singer T., Schuler F. (2018). A Sensitive In Vitro Approach to Assess the Hybridization-Dependent Toxic Potential of High Affinity Gapmer Oligonucleotides. Mol. Ther. Nucleic Acids.

[207] Burel S.A., Hart C.E., Cauntay P., Hsiao J., Machemer T., Katz M., Watt A., Bui H.H., Younis H., Sabripour M. (2016). Hepatotoxicity of high affinity gapmer antisense oligonucleotides is mediated by RNase H1 dependent promiscuous reduction of very long pre-mRNA transcripts. Nucleic Acids Res..

[208] Higuchi Y., Kawakami S., Fumoto S., Yamashita F., Hashida M. (2006). Effect of the particle size of galactosylated lipoplex on hepatocyte-selective gene transfection after intraportal administration. Biol. Pharm. Bull..

[209] Kawakami S., Ito Y., Fumoto S., Yamashita F., Hashida M. (2005). Enhanced gene expression in lung by a stabilized lipoplex using sodium chloride for complex formation. J. Gene Med..

[210] Ogawa K., Fuchigami Y., Hagimori M., Fumoto S., Miura Y., Kawakami S. (2018). Efficient gene transfection to the brain with ultrasound irradiation in mice using stabilized bubble lipopolyplexes prepared by the surface charge regulation method. Int. J. Nanomed..

[211] Kim J.K., Choi S.H., Kim C.O., Park J.S., Ahn W.S., Kim C.K. (2003). Enhancement of polyethylene glycol (PEG)-modified cationic liposome-mediated gene deliveries: Effects on serum stability and transfection efficiency. J. Pharm. Pharmacol..

[212] Balbino T.A., Serafin J.M., Malfatti-Gasperini A.A., de Oliveira C.L., Cavalcanti L.P., de Jesus M.B., de La Torre L.G. (2016). Microfluidic Assembly of pDNA/Cationic Liposome Lipoplexes with High pDNA Loading for Gene Delivery. Langmuir.

[213] Leung A.K., Tam Y.Y., Chen S., Hafez I.M., Cullis P.R. (2015). Microfluidic Mixing: A General Method for Encapsulating Macromolecules in Lipid Nanoparticle Systems. J. Phys. Chem. B.

[214] Lee R.J., Huang L. (1996). Folate-targeted, anionic liposome-entrapped polylysine-condensed DNA for tumor cell-specific gene transfer. J. Biol. Chem..

[215] Kurosaki T., Kitahara T., Fumoto S., Nishida K., Nakamura J., Niidome T., Kodama Y., Nakagawa H., To H., Sasaki H. (2009). Ternary complexes of pDNA, polyethylenimine, and gamma-polyglutamic acid for gene delivery systems. Biomaterials.

[216] Yamamoto T., Yahara A., Waki R., Yasuhara H., Wada F., Harada-Shiba M., Obika S. (2015). Amido-bridged nucleic acids with small hydrophobic residues enhance hepatic tropism of antisense oligonucleotides in vivo. Org. Biomol. Chem..

[217] Gaus H., Miller C.M., Seth P.P., Harris E.N. (2018). Structural Determinants for the Interactions of Chemically Modified Nucleic Acids with the Stabilin-2 Clearance Receptor. Biochemistry.

[218] Manoharan M., Inamati G.B., Lesnik E.A., Sioufi N.B., Freier S.M. (2002). Improving antisense oligonucleotide binding to human serum albumin: Dramatic effect of ibuprofen conjugation. Chembiochem.

[219] Hatakeyama H., Akita H., Ito E., Hayashi Y., Oishi M., Nagasaki Y., Danev R., Nagayama K., Kaji N., Kikuchi H. (2011). Systemic delivery of siRNA to tumors using a lipid nanoparticle containing a tumor-specific cleavable PEG-lipid. Biomaterials.

[220] Hayashi J.Y., Tamanoi F. (2017). Exploiting Enzyme Alterations in Cancer for Drug Activation, Drug Delivery, and Nanotherapy. Enzymes.

[221] Kozma G.T., Shimizu T., Ishida T., Szebeni J. (2020). Anti-PEG antibodies: Properties, formation, testing and role in adverse immune reactions to PEGylated nano-biopharmaceuticals. Adv. Drug Deliv. Rev..

[222] Hong L., Wang Z., Wei X., Shi J., Li C. (2020). Antibodies against polyethylene glycol in human blood: A literature review. J. Pharmacol. Toxicol. Methods.

[223] Fang J.-L., Beland F.A., Tang Y., Roffler S.R. (2020). Flow cytometry analysis of anti-polyethylene glycol antibodies in human plasma. Toxicol. Rep..

[224] Ishihara T., Maeda T., Sakamoto H., Takasaki N., Shigyo M., Ishida T., Kiwada H., Mizushima Y., Mizushima T. (2010). Evasion of the Accelerated Blood Clearance Phenomenon by Coating of Nanoparticles with Various Hydrophilic Polymers. Biomacromolecules.

[225] Abu Lila A.S., Uehara Y., Ishida T., Kiwada H. (2014). Application of Polyglycerol Coating to Plasmid DNA Lipoplex for the Evasion of the Accelerated Blood Clearance Phenomenon in Nucleic Acid Delivery. J. Pharm. Sci..

[226] Zou Y., Ito S., Yoshino F., Suzuki Y., Zhao L., Komatsu N. (2020). Polyglycerol Grafting Shields Nanoparticles from Protein Corona Formation to Avoid Macrophage Uptake. ACS Nano.

[227] Son K., Ueda M., Taguchi K., Maruyama T., Takeoka S., Ito Y. (2020). Evasion of the accelerated blood clearance phenomenon by polysarcosine coating of liposomes. J. Control. Release.

[228] Fumoto S., Kawakami S., Hashida M., Nishida K., Wei M., Good D. (2013). Capter 1. Targeted Gene Delivery: Importance of Administration Routes. Novel Gene Therapy Approaches.

[229] Kawabata K., Takakura Y., Hashida M. (1995). The fate of plasmid DNA after intravenous injection in mice: Involvement of scavenger receptors in its hepatic uptake. Pharm Res..

[230] Yang J. (2000). Intratumoral injection of naked DNA. Methods Mol. Med..

[231] Fumoto S., Nishi J., Nakamura J., Nishida K. (2008). Gene therapy for gastric diseases. Curr. Gene Ther..

[232] Peter G., Katrin W., Niclas C., Dinko R., Catarina N., Jane K., Mikko H., Yanfeng W., Rosie Y., Stanley R. (2020). Abstract 13307: An Oral Antisense Oligonucleotide for PCSK9 Inhibition in Humans. Circulation.

[233] Advances in RNAi Therapeutics Platform. https://www.alnylam.com/wp-content/uploads/2019/06/2019.06.22_Platform-Advances_FINAL.pdf.

[234] Raoof A.A., Ramtoola Z., McKenna B., Yu R.Z., Hardee G., Geary R.S. (2002). Effect of sodium caprate on the intestinal absorption of two modified antisense oligonucleotides in pigs. Eur. J. Pharm. Sci..

[235] Tillman L.G., Geary R.S., Hardee G.E. (2008). Oral Delivery of Antisense Oligonucleotides in Man. J. Pharm. Sci..

[236] Kreiter S., Selmi A., Diken M., Koslowski M., Britten C.M., Huber C., Türeci Ö., Sahin U. (2010). Intranodal Vaccination with Naked Antigen-Encoding RNA Elicits Potent Prophylactic and Therapeutic Antitumoral Immunity. Cancer Res..

[237] Ita K. (2017). Dermal/transdermal delivery of small interfering RNA and antisense oligonucleotides- advances and hurdles. Biomed. Pharmacother..

[238] Koh K.J., Liu Y., Lim S.H., Loh X.J., Kang L., Lim C.Y., Phua K.K.L. (2018). Formulation, characterization and evaluation of mRNA-loaded dissolvable polymeric microneedles (RNApatch). Sci. Rep..

[239] Yang T., Huang D., Li C., Zhao D., Li J., Zhang M., Chen Y., Wang Q., Liang Z., Liang X.-J. (2021). Rolling microneedle electrode array (RoMEA) empowered nucleic acid delivery and cancer immunotherapy. Nano Today.

[240] Boado R.J. (2007). Blood-brain barrier transport of non-viral gene and RNAi therapeutics. Pharm. Res..

[241] Danialou G., Comtois A.S., Matecki S., Nalbantoglu J., Karpati G., Gilbert R., Geoffroy P., Gilligan S., Tanguay J.F., Petrof B.J. (2005). Optimization of regional intraarterial naked DNA-mediated transgene delivery to skeletal muscles in a large animal model. Mol. Ther..

[242] Song K.H., Fan A.C., Hinkle J.J., Newman J., Borden M.A., Harvey B.K. (2017). Microbubble gas volume: A unifying dose parameter in blood-brain barrier opening by focused ultrasound. Theranostics.

[243] Negishi Y., Yamane M., Kurihara N., Endo-Takahashi Y., Sashida S., Takagi N., Suzuki R., Maruyama K. (2015). Enhancement of Blood-Brain Barrier Permeability and Delivery of Antisense Oligonucleotides or Plasmid DNA to the Brain by the Combination of Bubble Liposomes and High-Intensity Focused Ultrasound. Pharmaceutics.

[244] Oyama N., Fuchigami Y., Fumoto S., Sato M., Hagimori M., Shimizu K., Kawakami S. (2017). Characterization of transgene expression and pDNA distribution of the suctioned kidney in mice. Drug Deliv..

[245] Hattori Y., Kawakami S., Suzuki S., Yamashita F., Hashida M. (2004). Enhancement of immune responses by DNA vaccination through targeted gene delivery using mannosylated cationic liposome formulations following intravenous administration in mice. Biochem. Biophys. Res. Commun..

[246] Hattori Y., Kawakami S., Nakamura K., Yamashita F., Hashida M. (2006). Efficient gene transfer into macrophages and dendritic cells by in vivo gene delivery with mannosylated lipoplex via the intraperitoneal route. J. Pharmacol. Exp. Ther..

[247] Yamamoto T., Sawamura M., Terada C., Kashiwada K., Wada F., Yamayoshi A., Obika S., Harada-Shiba M. (2020). Effect of modular conjugation strategy for N-acetylgalactosamine-targeted antisense oligonucleotides. Nucleosides Nucleotides Nucleic Acids..

[248] Rejman J., Bragonzi A., Conese M. (2005). Role of clathrin- and caveolae-mediated endocytosis in gene transfer mediated by lipo- and polyplexes. Mol. Ther..

[249] Rejman J., Conese M., Hoekstra D. (2006). Gene transfer by means of lipo- and polyplexes: Role of clathrin and caveolae-mediated endocytosis. J. Liposome Res..

[250] Khalil I.A., Kogure K., Futaki S., Harashima H. (2006). High density of octaarginine stimulates macropinocytosis leading to efficient intracellular trafficking for gene expression. J. Biol. Chem..

[251] Fumoto S., Nishi J., Ishii H., Wang X., Miyamoto H., Yoshikawa N., Nakashima M., Nakamura J., Nishida K. (2009). Rac-mediated macropinocytosis is a critical route for naked plasmid DNA transfer in mice. Mol. Pharm..

[252] Takeuchi T., Futaki S. (2016). Current Understanding of Direct Translocation of Arginine-Rich Cell-Penetrating Peptides and Its Internalization Mechanisms. Chem. Pharm. Bull..

[253] Lehto T., Ezzat K., Wood M.J.A., El Andaloussi S. (2016). Peptides for nucleic acid delivery. Adv. Drug Deliv. Rev..

[254] Kuang H., Ku S.H., Kokkoli E. (2017). The design of peptide-amphiphiles as functional ligands for liposomal anticancer drug and gene delivery. Adv. Drug Deliv. Rev..

[255] Sugar I.P., Neumann E. (1984). Stochastic model for electric field-induced membrane pores. Electroporation. Biophys. Chem..

[256] Chang D.C., Reese T.S. (1990). Changes in membrane structure induced by electroporation as revealed by rapid-freezing electron microscopy. Biophys. J..

[257] Hu Y., Wan J.M., Yu A.C. (2013). Membrane perforation and recovery dynamics in microbubble-mediated sonoporation. Ultrasound Med. Biol..

[258] Zhang G., Gao X., Song Y.K., Vollmer R., Stolz D.B., Gasiorowski J.Z., Dean D.A., Liu D. (2004). Hydroporation as the mechanism of hydrodynamic delivery. Gene Ther..

[259] Mine T., Ishii H., Nakajima S., Yoshikawa N., Miyamoto H., Nakashima M., Nakamura J., Fumoto S., Nishida K. (2011). Rubbing gastric serosal surface enhances naked plasmid DNA transfer in rats and mice. Biol. Pharm. Bull..

[260] Fumoto S., Nakajima S., Mine T., Yoshikawa N., Kitahara T., Sasaki H., Miyamoto H., Nishida K. (2012). Efficient in vivo gene transfer by intraperitoneal injection of plasmid DNA and calcium carbonate microflowers in mice. Mol. Pharm..

[261] Durymanov M., Kamaletdinova T., Lehmann S.E., Reineke J.J. (2017). Exploiting passive nanomedicine accumulation at sites of enhanced vascular permeability for non-cancerous applications. J. Control. Release.

[262] Maeda H., Wu J., Sawa T., Matsumura Y., Hori K. (2000). Tumor vascular permeability and the EPR effect in macromolecular therapeutics: A review. J. Control. Release.

[263] Fang J., Nakamura H., Maeda H. (2011). The EPR effect: Unique features of tumor blood vessels for drug delivery, factors involved, and limitations and augmentation of the effect. Adv. Drug Deliv. Rev..

[264] Kimura N., Maeki M., Sato Y., Ishida A., Tani H., Harashima H., Tokeshi M. (2020). Development of a Microfluidic-Based Post-Treatment Process for Size-Controlled Lipid Nanoparticles and Application to siRNA Delivery. ACS Appl. Mater. Interfaces.

[265] Hashiba A., Toyooka M., Sato Y., Maeki M., Tokeshi M., Harashima H. (2020). The use of design of experiments with multiple responses to determine optimal formulations for in vivo hepatic mRNA delivery. J. Control. Release.

[266] Fumoto S., Nishida K. (2020). Co-delivery Systems of Multiple Drugs Using Nanotechnology for Future Cancer Therapy. Chem. Pharm. Bull..

[267] Kaps L., Schuppan D. (2020). Targeting Cancer Associated Fibroblasts in Liver Fibrosis and Liver Cancer Using Nanocarriers. Cells.

[268] Dolor A., Szoka F.C. (2018). Digesting a Path Forward: The Utility of Collagenase Tumor Treatment for Improved Drug Delivery. Mol. Pharm..

[269] Cemazar M., Golzio M., Sersa G., Escoffre J.-M., Coer A., Vidic S., Teissie J. (2012). Hyaluronidase and Collagenase Increase the Transfection Efficiency of Gene Electrotransfer in Various Murine Tumors. Hum. Gene Ther..

[270] Kato M., Hattori Y., Kubo M., Maitani Y. (2012). Collagenase-1 injection improved tumor distribution and gene expression of cationic lipoplex. Int. J. Pharm..

[271] Zinger A., Koren L., Adir O., Poley M., Alyan M., Yaari Z., Noor N., Krinsky N., Simon A., Gibori H. (2019). Collagenase Nanoparticles Enhance the Penetration of Drugs into Pancreatic Tumors. ACS Nano.

[272] Peng J.Q., Fumoto S., Suga T., Miyamoto H., Kuroda N., Kawakami S., Nishida K. (2019). Targeted co-delivery of protein and drug to a tumor in vivo by sophisticated RGD-modified lipid-calcium carbonate nanoparticles. J. Control. Release.

[273] Suga T., Fuchigami Y., Hagimori M., Kawakami S. (2017). Ligand peptide-grafted PEGylated liposomes using HER2 targeted peptide-lipid derivatives for targeted delivery in breast cancer cells: The effect of serine-glycine repeated peptides as a spacer. Int. J. Pharm..

[274] Bhattacharya S., Ghosh A., Maiti S., Ahir M., Debnath G.H., Gupta P., Bhattacharjee M., Ghosh S., Chattopadhyay S., Mukherjee P. (2020). Delivery of thymoquinone through hyaluronic acid-decorated mixed Pluronic® nanoparticles to attenuate angiogenesis and metastasis of triple-negative breast cancer. J. Control. Release.

[275] Rao N.V., Rho J.G., Um W., Ek P.K., Nguyen V.Q., Oh B.H., Kim W., Park J.H. (2020). Hyaluronic Acid Nanoparticles as Nanomedicine for Treatment of Inflammatory Diseases. Pharmaceutics.

[276] Hattab D., Bakhtiar A. (2020). Bioengineered siRNA-Based Nanoplatforms Targeting Molecular Signaling Pathways for the Treatment of Triple Negative Breast Cancer: Preclinical and Clinical Advancements. Pharmaceutics.

[277] Yang M., Li J., Gu P., Fan X. (2020). The application of nanoparticles in cancer immunotherapy: Targeting tumor microenvironment. Bioact. Mater..

[278] Kono Y., Kawakami S., Higuchi Y., Maruyama K., Yamashita F., Hashida M. (2014). Antitumor effect of nuclear factor-κB decoy transfer by mannose-modified bubble lipoplex into macrophages in mouse malignant ascites. Cancer Sci..

[279] Zhou Y. (2013). Ultrasound-Mediated Drug/Gene Delivery in Solid Tumor Treatment. J. Healthc. Eng..

[280] Fumoto S., Kawakami S. (2014). Combination of Nanoparticles with Physical Stimuli toward Cancer Therapy. Biol. Pharm. Bull..

[281] Lee S., Han H., Koo H., Na J.H., Yoon H.Y., Lee K.E., Lee H., Kim H., Kwon I.C., Kim K. (2017). Extracellular matrix remodeling in vivo for enhancing tumor-targeting efficiency of nanoparticle drug carriers using the pulsed high intensity focused ultrasound. J. Control. Release.

[282] Akinc A., Thomas M., Klibanov A.M., Langer R. (2005). Exploring polyethylenimine-mediated DNA transfection and the proton sponge hypothesis. J. Gene Med..

[283] Ukawa M., Akita H., Masuda T., Hayashi Y., Konno T., Ishihara K., Harashima H. (2010). 2-Methacryloyloxyethyl phosphorylcholine polymer (MPC)-coating improves the transfection activity of GALA-modified lipid nanoparticles by assisting the cellular uptake and intracellular dissociation of plasmid DNA in primary hepatocytes. Biomaterials.

[284] Tanaka H., Sato Y., Harashima H., Akita H. (2016). Cellular environment-responsive nanomaterials for use in gene and siRNA delivery: Molecular design for biomembrane destabilization and intracellular collapse. Expert Opin. Drug Deliv..

[285] Zanta M.A., Belguise-Valladier P., Behr J.P. (1999). Gene delivery: A single nuclear localization signal peptide is sufficient to carry DNA to the cell nucleus. Proc. Natl. Acad. Sci. USA.

[286] Miller A.M., Dean D.A. (2008). Cell-specific nuclear import of plasmid DNA in smooth muscle requires tissue-specific transcription factors and DNA sequences. Gene Ther..

[287] Gottfried L., Lin X., Barravecchia M., Dean D.A. (2016). Identification of an alveolar type I epithelial cell-specific DNA nuclear import sequence for gene delivery. Gene Ther..

[288] Tammam S.N., Azzazy H.M., Breitinger H.G., Lamprecht A. (2015). Chitosan Nanoparticles for Nuclear Targeting: The Effect of Nanoparticle Size and Nuclear Localization Sequence Density. Mol. Pharm..

[289] Xu Y., Liang W., Qiu Y., Cespi M., Palmieri G.F., Mason A.J., Lam J.K. (2016). Incorporation of a Nuclear Localization Signal in pH Responsive LAH4-L1 Peptide Enhances Transfection and Nuclear Uptake of Plasmid DNA. Mol. Pharm..

[290] Pazmany T., Murphy S.P., Gollnick S.O., Brooks S.P., Tomasi T.B. (1995). Activation of multiple transcription factors and fos and jun gene family expression in cells exposed to a single electric pulse. Exp. Cell Res..

[291] Nishikawa M., Nakayama A., Takahashi Y., Fukuhara Y., Takakura Y. (2008). Reactivation of silenced transgene expression in mouse liver by rapid, large-volume injection of isotonic solution. Hum. Gene Ther..

[292] Un K., Kawakami S., Higuchi Y., Suzuki R., Maruyama K., Yamashita F., Hashida M. (2011). Involvement of activated transcriptional process in efficient gene transfection using unmodified and mannose-modified bubble lipoplexes with ultrasound exposure. J. Control. Release.

[293] Mukai H., Kawakami S., Takahashi H., Satake K., Yamashita F., Hashida M. (2010). Key physiological phenomena governing transgene expression based on tissue pressure-mediated transfection in mice. Biol. Pharm. Bull..

[294] Haraguchi A., Fuchigami Y., Kawaguchi M., Fumoto S., Ohyama K., Shimizu K., Hagimori M., Kawakami S. (2018). Determining Transgene Expression Characteristics Using a Suction Device with Multiple Hole Adjusting a Left Lateral Lobe of the Mouse Liver. Biol. Pharm. Bull..

[295] Yew N.S., Zhao H., Przybylska M., Wu I.H., Tousignant J.D., Scheule R.K., Cheng S.H. (2002). CpG-depleted plasmid DNA vectors with enhanced safety and long-term gene expression in vivo. Mol. Ther..

[296] Wong S.P., Argyros O., Coutelle C., Harbottle R.P. (2011). Non-viral S/MAR vectors replicate episomally in vivo when provided with a selective advantage. Gene Ther..

[297] Nishimura K., Fumoto S., Fuchigami Y., Hagimori M., Maruyama K., Kawakami S. (2017). Effective intraperitoneal gene transfection system using nanobubbles and ultrasound irradiation. Drug Deliv..

[298] Nakanishi H., Higuchi Y., Kawakami S., Yamashita F., Hashida M. (2010). piggyBac transposon-mediated long-term gene expression in mice. Mol. Ther..

[299] Otani Y., Kawakami S., Mukai H., Fuchigami Y., Yamashita F., Hashida M. (2015). Long-term in vivo gene expression in mouse kidney using φC31 integrase and electroporation. J. Drug Target..

[300] Miller J.B., Zhang S., Kos P., Xiong H., Zhou K., Perelman S.S., Zhu H., Siegwart D.J. (2017). Non-Viral CRISPR/Cas Gene Editing In Vitro and In Vivo Enabled by Synthetic Nanoparticle Co-Delivery of Cas9 mRNA and sgRNA. Angew. Chem. Int. Ed..

[301] Nair J.K., Attarwala H., Sehgal A., Wang Q., Aluri K., Zhang X., Gao M., Liu J., Indrakanti R., Schofield S. (2017). Impact of enhanced metabolic stability on pharmacokinetics and pharmacodynamics of GalNAc–siRNA conjugates. Nucleic Acids Res..

[302] Ray K.K., Landmesser U., Leiter L.A., Kallend D., Dufour R., Karakas M., Hall T., Troquay R.P., Turner T., Visseren F.L. (2017). Inclisiran in Patients at High Cardiovascular Risk with Elevated LDL Cholesterol. N. Engl. J. Med..

[303] Brown C.R., Gupta S., Qin J., Racie T., He G., Lentini S., Malone R., Yu M., Matsuda S., Shulga-Morskaya S. (2020). Investigating the pharmacodynamic durability of GalNAc-siRNA conjugates. Nucleic Acids Res..

[304] Shen W., De Hoyos C.L., Migawa M.T., Vickers T.A., Sun H., Low A., Bell T.A., Rahdar M., Mukhopadhyay S., Hart C.E. (2019). Chemical modification of PS-ASO therapeutics reduces cellular protein-binding and improves the therapeutic index. Nat. Biotechnol..

[305] Griesenbach U., Meng C., Farley R., Gardner A., Brake M.A., Frankel G.M., Gruenert D.C., Cheng S.H., Scheule R.K., Alton E.W. (2009). The role of doxorubicin in non-viral gene transfer in the lung. Biomaterials.

[306] Un K., Kono Y., Yoshida M., Yamashita F., Kawakami S., Hashida M. (2012). Enhancement of gene expression by transcriptional activation using doxorubicin-loaded liposome/pDNA complexes. Pharmazie.

[307] Wang S., Fumoto S., Miyamoto H., Tanaka M., Nishida K. (2018). Edaravone, a cytoprotective drug, enhances transgene expression mediated by lipoplexes in HepG2 cells and mice. Int. J. Pharm..

[308] Takiguchi N., Takahashi Y., Nishikawa M., Matsui Y., Fukuhara Y., Oushiki D., Kiyose K., Hanaoka K., Nagano T., Takakura Y. (2011). Positive correlation between the generation of reactive oxygen species and activation/reactivation of transgene expression after hydrodynamic injections into mice. Pharm. Res..

[309] Fumoto S., Nishida K. (2017). Methods for Evaluating the Stimuli-Responsive Delivery of Nucleic Acid and Gene Medicines. Chem. Pharm. Bull..

[310] Kuchimaru T., Iwano S., Kiyama M., Mitsumata S., Kadonosono T., Niwa H., Maki S., Kizaka-Kondoh S. (2016). A luciferin analogue generating near-infrared bioluminescence achieves highly sensitive deep-tissue imaging. Nat. Commun..

[311] Fumoto S., Nishimura K., Nishida K., Kawakami S. (2016). Three-Dimensional Imaging of the Intracellular Fate of Plasmid DNA and Transgene Expression: ZsGreen1 and Tissue Clearing Method CUBIC Are an Optimal Combination for Multicolor Deep Imaging in Murine Tissues. PLoS ONE.

[312] Fumoto S., Kinoshita E., Ohta K., Nakamura K.I., Hirayama T., Nagasawa H., Hu D., Okami K., Kato R., Shimokawa S. (2020). A pH-Adjustable Tissue Clearing Solution That Preserves Lipid Ultrastructures: Suitable Tissue Clearing Method for DDS Evaluation. Pharmaceutics.

[313] Hisazumi J., Kobayashi N., Nishikawa M., Takakura Y. (2004). Significant role of liver sinusoidal endothelial cells in hepatic uptake and degradation of naked plasmid DNA after intravenous injection. Pharm. Res..

[314] Shimizu K., Higuchi Y., Kozu Y., Hashida M., Konishi S. (2011). Development of a suction device for stabilizing in vivo real-time imaging of murine tissues. J. Biosci. Bioeng..

[315] Wang B.G., König K., Halbhuber K.J. (2010). Two-photon microscopy of deep intravital tissues and its merits in clinical research. J. Microsc..

[316] Fumoto S., Kawakami S., Ishizuka M., Nishikawa M., Yamashita F., Hashida M. (2003). Analysis of Hepatic Disposition of Native and Galactosylated Polyethylenimine Complexed with Plasmid DNA in Perfused Rat Liver. Drug Metab. Pharmacokinet..

[317] Fumoto S., Nakadori F., Kawakami S., Nishikawa M., Yamashita F., Hashida M. (2003). Analysis of hepatic disposition of galactosylated cationic liposome/plasmid DNA complexes in perfused rat liver. Pharm. Res..

[318] Ko Y.T. (2013). Nanoparticle-mediated delivery of oligonucleotides to the blood–brain barrier:in vitroandin situbrain perfusion studies on the uptake mechanisms. J. Drug Target..

[319] Minchin R.F., Johnston M.R., Aiken M.A., Boyd M.R. (1984). Pharmacokinetics of doxorubicin in isolated lung of dogs and humans perfused in vivo. J. Pharmacol. Exp. Ther..

[320] Sawai K., Miyao T., Takakura Y., Hashida M. (1995). Renal Disposition Characteristics of Oligonucleotides Modified at Terminal Linkages in the Perfused Rat Kidney. Antisense Res. Dev..

[321] Kakutani T., Yamaoka K., Hashida M., Sezaki H. (1985). A new method for assessment of drug disposition in muscle: Application of statistical moment theory to local perfusion systems. J. Pharmacokinet. Biopharm..

[322] Nomura T., Nakajima S., Kawabata K., Yamashita F., Takakura Y., Hashida M. (1997). Intratumoral pharmacokinetics and in vivo gene expression of naked plasmid DNA and its cationic liposome complexes after direct gene transfer. Cancer Res..

[323] Akita H., Ito R., Khalil I.A., Futaki S., Harashima H. (2004). Quantitative three-dimensional analysis of the intracellular trafficking of plasmid DNA transfected by a nonviral gene delivery system using confocal laser scanning microscopy. Mol. Ther..

[324] Lambert T.J., Waters J.C. (2017). Navigating challenges in the application of superresolution microscopy. J. Cell Biol..

[325] Jiang Y., Lu Q., Wang Y., Xu E., Ho A., Singh P., Wang Y., Jiang Z., Yang F., Tietjen G.T. (2020). Quantitating Endosomal Escape of a Library of Polymers for mRNA Delivery. Nano Lett..

[326] Zhang Y., Qu Z., Kim S., Shi V., Liao B., Kraft P., Bandaru R., Wu Y., Greenberger L.M., Horak I.D. (2011). Down-modulation of cancer targets using locked nucleic acid (LNA)-based antisense oligonucleotides without transfection. Gene Ther..

[327] Stein C.A., Hansen J.B., Lai J., Wu S., Voskresenskiy A., Høg A., Worm J., Hedtjärn M., Souleimanian N., Miller P. (2010). Efficient gene silencing by delivery of locked nucleic acid antisense oligonucleotides, unassisted by transfection reagents. Nucleic Acids Res..

[328] Hori S.-I., Yamamoto T., Waki R., Wada S., Wada F., Noda M., Obika S. (2015). Ca^2+^ enrichment in culture medium potentiates effect of oligonucleotides. Nucleic Acids Res..

[329] Matsuura S., Katsumi H., Suzuki H., Hirai N., Takashima R., Morishita M., Sakane T., Yamamoto A. (2018). l-Cysteine and l-Serine Modified Dendrimer with Multiple Reduced Thiols as a Kidney-Targeting Reactive Oxygen Species Scavenger to Prevent Renal Ischemia/Reperfusion Injury. Pharmaceutics.

[330] Yamashita S., Katsumi H., Shimizu E., Nakao Y., Yoshioka A., Fukui M., Kimura H., Sakane T., Yamamoto A. (2020). Dendrimer-based micelles with highly potent targeting to sites of active bone turnover for the treatment of bone metastasis. Eur. J. Pharm. Biopharm..

[331] Nishimura K., Yonezawa K., Fumoto S., Miura Y., Hagimori M., Nishida K., Kawakami S. (2019). Application of Direct Sonoporation from a Defined Surface Area of the Peritoneum: Evaluation of Transfection Characteristics in Mice. Pharmaceutics.

[332] Endo-Takahashi Y., Negishi Y. (2020). Microbubbles and Nanobubbles with Ultrasound for Systemic Gene Delivery. Pharmaceutics.

[333] Negishi Y., Endo-Takahashi Y., Ishiura S. (2018). Exon Skipping by Ultrasound-Enhanced Delivery of Morpholino with Bubble Liposomes for Myotonic Dystrophy Model Mice. Methods Mol. Biol..

[334] Taniguchi Y., Oyama N., Fumoto S., Kinoshita H., Yamashita F., Shimizu K., Hashida M., Kawakami S. (2020). Tissue suction-mediated gene transfer to the beating heart in mice. PLoS ONE.

[335] Lenk G.M., Jafar-Nejad P., Hill S.F., Huffman L.D., Smolen C.E., Wagnon J.L., Petit H., Yu W., Ziobro J., Bhatia K. (2020). Scn8a Antisense Oligonucleotide Is Protective in Mouse Models of SCN8A Encephalopathy and Dravet Syndrome. Ann. Neurol..

[336] Bennett C.F., Krainer A.R., Cleveland D.W. (2019). Antisense Oligonucleotide Therapies for Neurodegenerative Diseases. Annu. Rev. Neurosci..

[337] Zhang X., Goel V., Robbie G.J. (2019). Pharmacokinetics of Patisiran, the First Approved RNA Interference Therapy in Patients with Hereditary Transthyretin-Mediated Amyloidosis. J. Clin. Pharmacol..

[338] Chen C.-Y., Tran D.M., Cavedon A., Cai X., Rajendran R., Lyle M.J., Martini P.G., Miao C.H. (2020). Treatment of Hemophilia A Using Factor VIII Messenger RNA Lipid Nanoparticles. Mol. Ther. Nucleic Acids.

[339] Kim J., Eygeris Y., Gupta M., Sahay G. (2021). Self-assembled mRNA vaccines. Adv. Drug Deliv. Rev..

[340] Seraj S., Lee J., Ahn H.J. (2019). Systemic delivery of Eg5 shRNA-expressing plasmids using PEGylated DC-Chol/DOPE cationic liposome: Long-term silencing and anticancer effects in vivo. Biochem. Pharmacol..

[341] Bazzani R.P., Pringle I.A., Connolly M.M., Davies L.A., Sumner-Jones S.G., Schleef M., Hyde S.C., Gill D.R. (2016). Transgene sequences free of CG dinucleotides lead to high level, long-term expression in the lung independent of plasmid backbone design. Biomaterials.

[342] Ofri R., Averbukh E., Ezra-Elia R., Ross M., Honig H., Obolensky A., Rosov A., Hauswirth W.W., Gootwine E., Banin E. (2018). Six Years and Counting: Restoration of Photopic Retinal Function and Visual Behavior Following Gene Augmentation Therapy in a Sheep Model of CNGA3 Achromatopsia. Hum. Gene Ther..

[343] Al-Zaidy S.A., Mendell J.R. (2019). From Clinical Trials to Clinical Practice: Practical Considerations for Gene Replacement Therapy in SMA Type 1. Pediatr. Neurol..

[344] Verdera H.C., Kuranda K., Mingozzi F. (2020). AAV Vector Immunogenicity in Humans: A Long Journey to Successful Gene Transfer. Mol. Ther..

[345] Breunig M., Lungwitz U., Liebl R., Goepferich A. (2007). Breaking up the correlation between efficacy and toxicity for nonviral gene delivery. Proc. Natl. Acad. Sci. USA.

[346] Huang Q., Li S., Ding Y.-F., Yin H., Wang L.H., Wang R. (2018). Macrocycle-wrapped polyethylenimine for gene delivery with reduced cytotoxicity. Biomater. Sci..

[347] Iredale J.P. (2007). Models of liver fibrosis: Exploring the dynamic nature of inflammation and repair in a solid organ. J. Clin. Investig..

[348] Böttger R., Pauli G., Chao P.-H., Al Fayez N., Hohenwarter L., Li S.D. (2020). Lipid-based nanoparticle technologies for liver targeting. Adv. Drug Deliv. Rev..

[349] Niemietz C., Nadzemova O., Zibert A., Schmidt H.H.-J. (2020). APOE polymorphism in ATTR amyloidosis patients treated with lipid nanoparticle siRNA. Amyloid.

[350] Ghiselli G., Schaefer E.J., Gascon P., Breser H.B. (1981). Type III hyperlipoproteinemia associated with apolipoprotein E deficiency. Science.

[351] Tokunaga A., Miyamoto H., Fumoto S., Nishida K. (2019). Effect of renal ischaemia/reperfusion-induced acute kidney injury on pharmacokinetics of midazolam in rats. J. Pharm. Pharmacol..

[352] Tokunaga A., Miyamoto H., Fumoto S., Nishida K. (2020). Effect of Chronic Kidney Disease on Hepatic Clearance of Drugs in Rats. Biol. Pharm. Bull..

[353] Nakano T., Katsuki S., Chen M., Decano J.L., Halu A., Lee L.H., Pestana D.V.S., Kum A.S.T., Kuromoto R.K., Golden W.S. (2019). Uremic Toxin Indoxyl Sulfate Promotes Proinflammatory Macrophage Activation via the Interplay of OATP2B1 and Dll4-Notch Signaling. Circulation.

[354] Li Y., Yan J., Wang M., Lv J., Yan F., Chen J. (2021). Uremic toxin indoxyl sulfate promotes proinflammatory macrophage activation by regulation of β-catenin and YAP pathways. J. Mol. Histol..

[355] Nishimura K., Ogawa K., Kawaguchi M., Fumoto S., Mukai H., Kawakami S. (2021). Suppression of Peritoneal Fibrosis by Sonoporation of Hepatocyte Growth Factor Gene-Encoding Plasmid DNA in Mice. Pharmaceutics.

[356] Peng J., Fumoto S., Miyamoto H., Chen Y., Kuroda N., Nishida K. (2017). One-step formation of lipid-polyacrylic acid-calcium carbonate nanoparticles for co-delivery of doxorubicin and curcumin. J. Drug Target..

